# Age-associated callus senescent cells produce TGF-**β**1 that inhibits fracture healing in aged mice

**DOI:** 10.1172/JCI148073

**Published:** 2022-04-15

**Authors:** Jiatong Liu, Jun Zhang, Xi Lin, Brendan F. Boyce, Hengwei Zhang, Lianping Xing

**Affiliations:** 1 Department of Pathology and Laboratory Medicine, Center for Musculoskeletal Research, University of Rochester Medical Center, Rochester, New York, USA.; 2 Plastic Surgery Center, Department of Orthopedics, Zhejiang Provincial People’s Hospital, Hangzhou, Zhejiang, China.; 3 Suzhou Institute of Systems Medicine, Suzhou, China.

**Keywords:** Aging, Bone Biology, Bone disease, Cellular senescence, Osteoclast/osteoblast biology

## Abstract

Cellular senescence plays an important role in human diseases, including osteoporosis and osteoarthritis. Senescent cells (SCs) produce the senescence-associated secretory phenotype to affect the function of neighboring cells and SCs themselves. Delayed fracture healing is common in the elderly and is accompanied by reduced mesenchymal progenitor cells (MPCs). However, the contribution of cellular senescence to fracture healing in the aged has not to our knowledge been studied. Here, we used C57BL/6J 4-month-old young and 20-month-old aged mice and demonstrated a rapid increase in SCs in the fracture callus of aged mice. The senolytic drugs dasatinib plus quercetin enhanced fracture healing in aged mice. Aged callus SCs inhibited the growth and proliferation of callus-derived MPCs (CaMPCs) and expressed high levels of TGF-**β**1. TGF-**β**–neutralizing Ab prevented the inhibitory effects of aged callus SCs on CaMPCs and promoted fracture healing in aged mice, which was associated with increased CaMPCs and proliferating cells. Thus, fracture triggered a significant cellular senescence in the callus cells of aged mice, which inhibited MPCs by expressing TGF-**β**1. Short-term administration of dasatinib plus quercetin depleted callus SCs and accelerated fracture healing in aged mice. Senolytic drugs represent a promising therapy, while TGF-**β**1 signaling is a molecular mechanism for fractures in the elderly via SCs.

## Graphical abstract

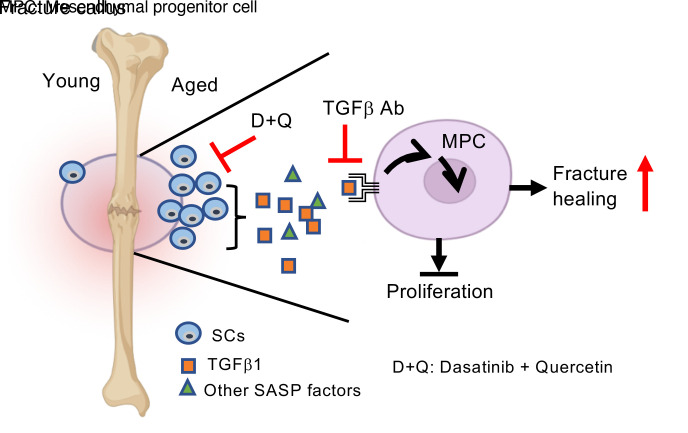

## Introduction

More than 5 million long bone fractures occur annually in the United States, and 15% of them occur in people older than 50 years of age (1). Furthermore, most elderly individuals have decreased healing potential (2, 3) and increased mortality following fracture due to complications (4). There are no FDA-approved drugs to enhance fracture healing in the elderly, nor are the molecular mechanisms that delay fracture healing in aged individuals well studied (5).

Fracture healing involves 4 stages, including the formation of a hematoma, a soft callus, a hard callus, and remodeling. The soft callus consists of internal and external components and occurs approximately 5 to 10 days after fracture in mice. The internal callus is produced by cells from the endosteum and is composed of a fibrocartilaginous matrix, whereas the external callus is produced by cells from the periosteum and consists of hyaline cartilage and bone (6). At this stage, mesenchymal progenitor cells (MPCs) are recruited to the fracture site, where they proliferate and give rise to osteochondrogenic cells. Josephson et al. reported recently that, following fracture, 12-month-old middle-aged mice have impaired healing, decreased MPC numbers, and increased senescence in MPCs induced by serum. Of note, this study used BM MPCs. Antiinflammatory drugs (i.e., NSAIDs) reduce the senescence-associated secretory phenotype (SASP), MPC senescence, and ultimately improve fracture healing (7). This study suggests that BM MPC senescence (caused by systemic inflammation) contributed to impaired fracture healing in middle-aged mice. However, it did not examine whether the senescent cells (SCs) within the callus affect fracture healing in aged mice or the mechanisms involved.

Cellular senescence is characterized by permanent cell proliferation arrest and an altered gene expression pattern, leading to the SASP (8, 9). The SASP comprises dysfunctional factors secreted by SCs, including growth factors, cytokines, chemokines, and proteases (10) and may also consist of exosomes, miRNA, and other bioactive factors. The SASP and inflammatory cytokines are not the same, but they have some overlapping effects. SCs produce SASP factors, forming a toxic microenvironment and affecting the functions of neighboring cells and SCs themselves, via autocrine/paracrine pathways. The SASP also contributes to the generation and accumulation of additional SCs (11).

Farr et al. reported high expression of numerous SASP factors in enriched cell populations isolated from bone or bone marrow of 24-month-old aged mice (10). We found that callus SCs of aged mice expressed higher levels of TGF-β1 than did SCs from young mice. TGF-β1 is one of the major SASP components (12, 13). The TGF-β superfamily has complex functions in bone cells due to temporal and spatial interactions among multiple ligands, receptors, and downstream signals, which are situation dependent (14). For example, a single injection of TGF-β1 plus IGF1 enhances healing of femoral defects in old rats (15). TGF-β receptor (TβR) kinase inhibitor impairs fracture healing in medaka fish by inhibiting the migration and differentiation of osteoblasts (OBs) (16). In contrast, Camurati-Engelmann disease is a sclerotic bone dysplasia caused by activating mutations in the *TGFB1* gene, which can be rescued by a bone-targeted TβR1 inhibitor (17). Bone samples from aged mice and humans have elevated TGF-β1 levels, and TGF-β1 inhibits MPC differentiation into OBs by promoting the degradation of TNF receptor–associated factor 3 to contribute to age-related osteoporosis (18). Patients with nonunion fractures have high levels of TGF-β1 in the bones and blood (19). However, the role of TGF-β in fracture repair in aged mice, especially its relation with cellular senescence of callus cells, has not been studied to date.

Aging is an intrinsic feature of living organisms and is accompanied by increased cellular senescence in multiple cell types (20–22). Removal of SCs by a genetic approach or senolytic drugs attenuates age-associated changes, including bone loss and osteoarthritis (OA) (23, 24). However, in addition to having detrimental effects, SCs may also be beneficial under certain conditions. SCs accelerate acute wound healing by inducing myofibroblast differentiation through the secretion of PDGF AA (25). In addition, tumor cell production of p53 triggers cellular senescence and the production of SASP factors, leading to tumor cell clearance (26). Interestingly, fracture in aged bones represents a special callus tissue microenvironment that combines acute injury and natural aging, which has not been well studied. Two recent reports studied cellular senescence in fracture healing. Khosla et al. showed that senolytic drugs enhance fracture healing in young adult mice (27). Ding et al. reported that middle-aged *p16*-knockout mice have accelerated fracture healing (28). However, whether SCs mediate this effect of *p16* deficiency on fracture healing or whether clearance of SCs affects fracture healing in young and aged mice differently is not known.

Here, we used 4-month-old young and 20-month-old aged mice to determine whether SCs contribute to fracture healing in aged mice and the molecular mechanisms involved. Our overall hypothesis is that in the callus of aged mice, SCs produce excessive TGF-β1, which inhibits the proliferation of callus MPCs and delays fracture healing, an effect that can be prevented by TGF-β neutralization. We found that SCs increased rapidly in aged callus and expressed TGF-β1. High levels of TGF-β1 persisted in aged mice. Conditioned medium collected from aged callus inhibited MPC growth and proliferation, which was prevented by a TGF-β–neutralizing Ab. Inhibition of TGF-β signaling markedly enhanced fracture healing in aged mice, which was accompanied by an increase in callus MPCs and cell proliferation. Thus, fracture triggers cellular senescence in the callus of aged mice via TGF-β1 signaling, which is a potential molecular mechanism to inhibit fracture repair in aging mice.

## Results

### SCs are increased in the fracture callus of aged mice.

**Figure 1:**
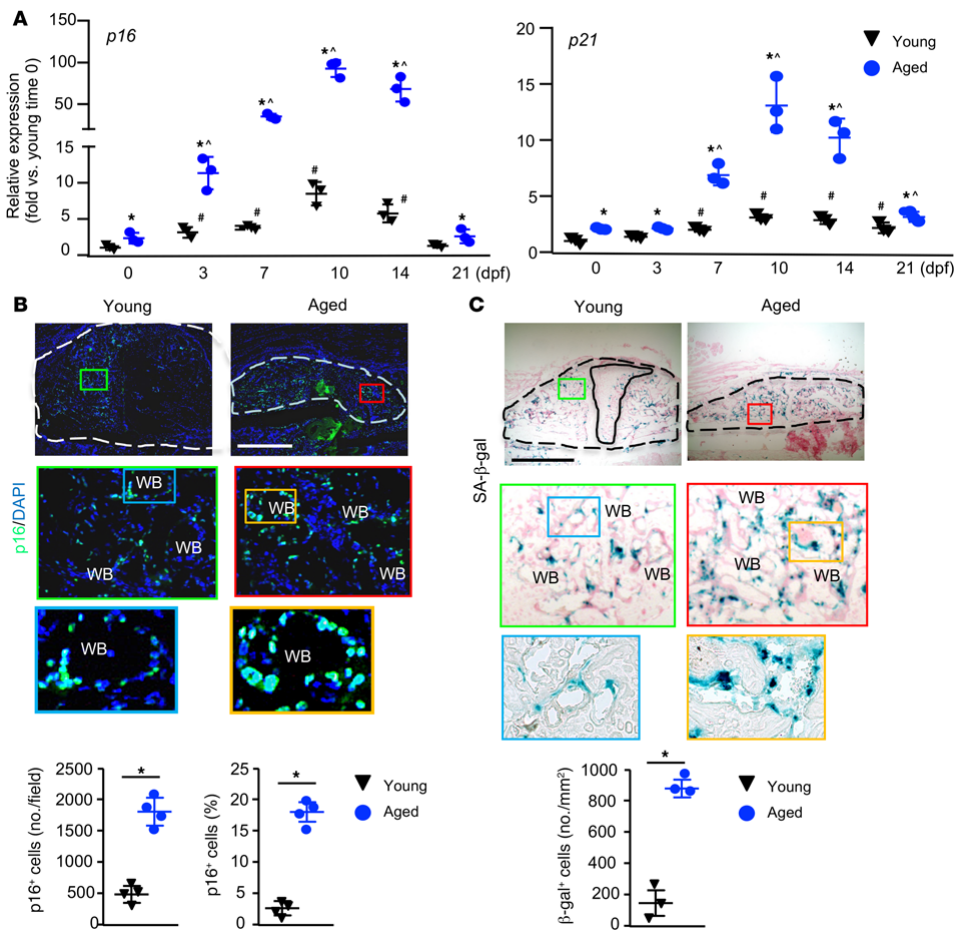
Increase in p16^+^ SCs in fracture callus of aged mice. Young and aged mice underwent tibial fracture surgery and were sacrificed at 10 dpf. (**A**) Expression of *p16* and *p21* in callus tissue was measured by qPCR at the indicated time points. *n =* 3. Relative mRNA expression is the fold change versus young samples at time 0 as 1. *P < 0.05 aged versus young; ^#^*P <* 0.05 young versus young 0 dpf; and ^*P <* 0.05 aged versus aged 0 dpf, by 2-way ANOVA followed by Tukey’s post hoc test. (**B** and **C**) Frozen sections of callus were immunostained with anti-p16 Ab or SA–β-gal. External callus is indicated by a dashed line and cartilage by a solid line. WB, woven bone. (**B**) Representative images showing increased p16^+^ cells in the aged sample. Scale bar: 1 mm. The number and percentage of p16^+^ cells were quantified by ImageJ. *n =* 4. (**C**) Representative images showing increased SA–β-gal^+^ cells in the aged sample. Scale bar: 1 mm. Original magnification, ×7 (enlarged insets in **B**) and ×3.5 (enlarged insets in **C**). SA–β-gal^+^ cells were quantified by ImageJ. *n =* 3. *P < 0.05, by unpaired, 2-tailed Student’s *t* test.

SCs express high levels of senescence effector genes in 2 major senescence pathways, including the p16/pRB and p19/p53/p21 pathways. p16^Ink4a^ (p16) and p21^Cip1^ (p21) are 2 well-established senescence markers and act as cell cycle inhibitors by blocking progression through G_1_/S (29–31). We measured the expression levels of *p16* and *p21* in callus tissues collected at different time points following fracture and from nonfractured bone samples collected at time 0 as a control. We used these as outcome measures for the presence of SCs during the course of fracture healing. In both young and aged mice, the expression of *p16* and *p21* increased following fracture compared with nonfractured bone samples (samples at time 0), peaked on day 10, and declined thereafter. Aged mice expressed higher levels of *p16* and *p21* at most time points than did young mice at the same time points (Figure 1A). Levels of *p18* and *p19*, other senescence-associated genes (22, 32), were also higher in aged callus (Supplemental Figure 1; supplemental material available online with this article; https://doi.org/10.1172/JCI148073DS1). Using immunohistochemistry with an anti-p16 Ab, we found that the numbers of p16^+^ SCs increased significantly in the callus of aged mice compared with that seen in young mice (Figure 1B). This was confirmed by histochemical staining for senescence-associated β-gal (SA–β-gal), a widely used marker for SCs (Figure 1C). We did not identify SCs in callus cartilage. The distribution pattern of SCs in the callus area was similar in the young and aged mice. To confirm the specificity of the p16 Ab, we treated callus MPCs from *p16^tdTom/tdTom^* (*p16*-deficient mice) and WT control mice (which do not carry the *p16^tdTom^* allele) with H_2_O_2_ to induce cellular senescence. WT cells stained positively, whereas *p16^tdTom/tdTom^* cells stained negatively with the anti-p16 Ab. At the same time, both the WT and *p16^tdTom/tdTom^* cells stained positively for SA–β-gal, indicating that they were SCs. As another negative control, WT cells stained negatively when only the secondary Ab was used (Supplemental Figure 2).

**Figure 2:**
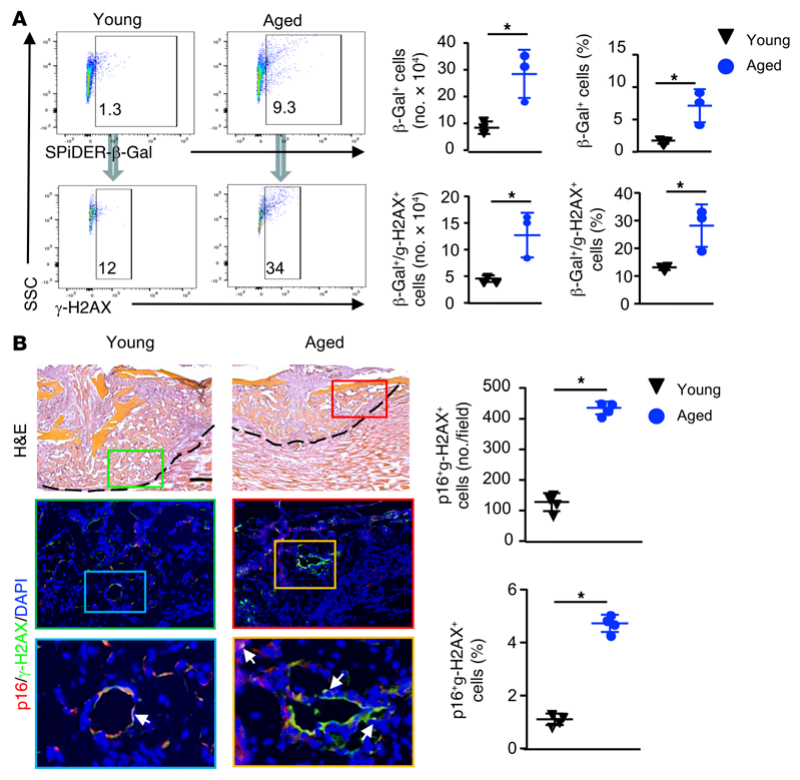
Increase in γ-H2AX^+^ SCs in fracture callus of aged mice. Young and aged mice were sacrificed at 10 dpf. (**A**) Cells from callus tissues were subjected to flow cytometry to detect SPiDER–β-gal and γ-H2AX. Quantification of the number and percentage of SPiDER–β-gal^+^, γ-H2AX^+^, and SPiDER–β-gal^+^γ-H2AX^+^ cells. *n =* 3. The numbers in the plots indicate the percentage of positive cells from a representative sample. *P < 0.05, by unpaired, 2-tailed Student’s *t* test. (**B**) Frozen sections of callus immunostained with p16 or γ-H2AX Ab. H&E-stained images (upper panels) indicate the location of the enlarged area in the immunostained sections (middle and lower panels). Scale bar: 250 μm. Original magnification, ×3 (enlarged insets). p16 and γ-H2AX double-positive cells are indicated by arrows. The number and percentage of p16^+^γ-H2AX^+^ cells were quantified by ImageJ. *n =* 4. *P < 0.05, by unpaired, 2-tailed Student’s *t* test.

To further characterize callus SCs, we used γ-H2AX staining, because SCs are often associated with DNA damage, which can be identified by γ-H2AX^+^ staining (33). We first performed flow cytometry using cells directly isolated from callus tissues from young and aged mice for β-gal and γ-H2AX expression. We found that callus cells from aged mice had significantly more β-gal^+^ cells and γ-H2AX^+^ cells than did those from young mice. In aged callus cells, approximately 35% of the β-gal^+^ cells were γ-H2AX^+^, and the number and percentage of β-gal^+^γ-H2AX^+^ cells were higher than in young callus cells (Figure 2A). We also performed double-immunostaining on decalcified callus sections with p16 and γ-H2AX Abs and found that approximately 30% of p16^+^ cells stained positively for γ-H2AX (Figure 2B, arrows). Similar to the data from the flow cytometric analysis in Figure 2A, we detected significantly more p16/γ-H2AX double-positive cells in the callus of aged mice than in that of young mice (Figure 2B).

### Clearance of SCs enhances fracture repair in aged mice.

**Figure 3:**
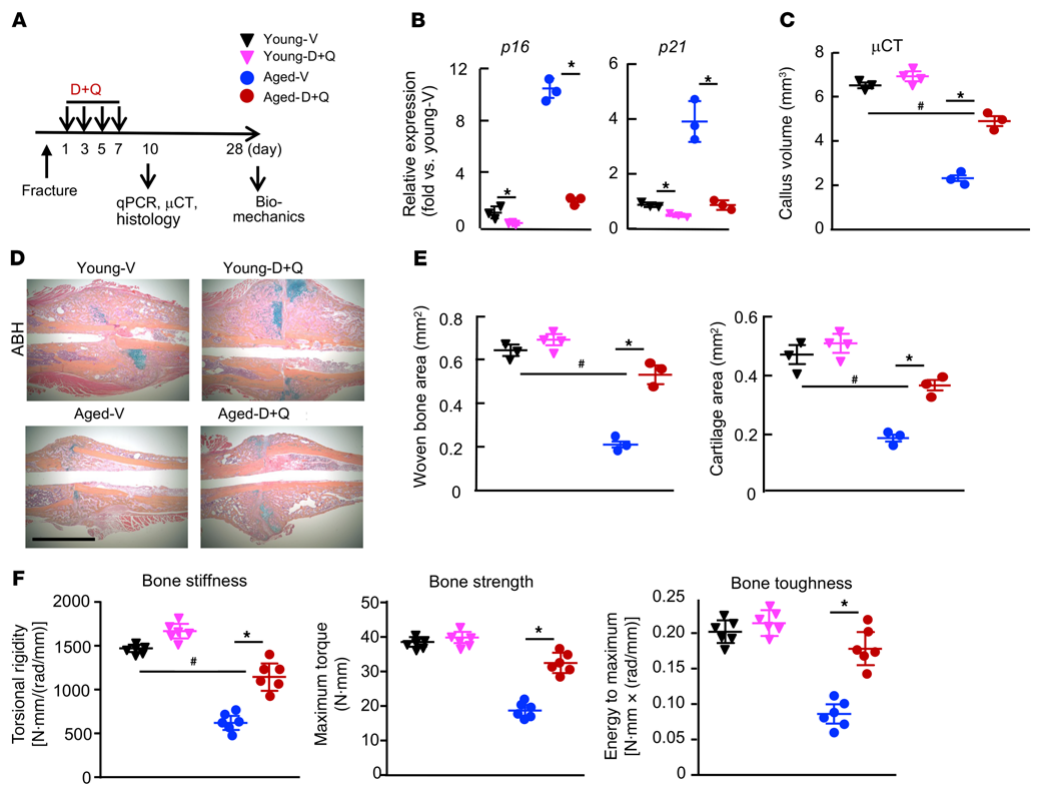
Senolytic drugs enhance fracture healing in aged mice by removing SCs. Young and aged mice underwent tibial fracture surgery. (**A**) Experimental outline. Young and aged mice were given 5 mg/kg dasatinib (D) + 50 mg/kg quercetin (Q) or vehicle (V) by gavage on 1, 3, 5, and 7 dpf and sacrificed at 10 or 28 dpf. (**B**) Expression of *p16* and *p21* in callus tissues was measured by qPCR at 10 dpf. *n =* 3. Relative mRNA expression is the fold change versus vehicle-treated young mice as 1. *P < 0.05 vehicle versus D+Q, by 2-way ANOVA followed by Tukey’s post hoc test. (**C**) Callus volume was measured by micro-CT. *n =* 3–4. (**D**) Representative ABH-stained sections showing higher woven bone and callus areas in D+Q-treated mice. Scale bar: 1 mm. (**E**) Woven bone and cartilage areas were analyzed by ImageJ. *n =* 3–4. (**F**) Bone stiffness, strength, and toughness were assessed by biomechanical testing at 28 dpf. *n =* 6. *P < 0.05 vehicle versus D+Q treatment; ^#^*P <* 0.05, for D+Q-treated aged mice versus vehicle-treated young mice; 2-way ANOVA followed by Tukey’s post hoc test (**C**, **E**, and **F**). μCT, micro-CT.

To determine whether inhibition of SCs could promote fracture healing in aged mice, we treated young and aged mice with dasatinib and quercetin (D+Q), senolytic drugs that kill SCs (23, 34). We chose to treat mice with D+Q at 1, 3, 5, and 7 days post fracture (dpf), because we aimed to deplete SCs before they reached their peak in order to block the detrimental effect of SC-produced SASP factors on MPCs. We found markedly increased expression of senescence marker genes in fracture callus starting on 3 dpf and peaking on 10 dpf (Figure 1A). Further, we and others have reported that administration of bone anabolic agents at the early phase of fracture healing, the same protocol that we used here, could promote fracture healing by increasing MPCs (35, 36). This treatment reduced the expression in callus of senescence genes, which associated with increased cartilage, new bone area, and bone strength only in aged mice (Figure 3 and Supplemental Figure 3). These findings suggest that accumulation of SCs in the early post-fracture period contributed to delayed fracture repair in aged mice.

### SCs from aged callus inhibit the growth and proliferation of callus-derived MPCs.

**Figure 4:**
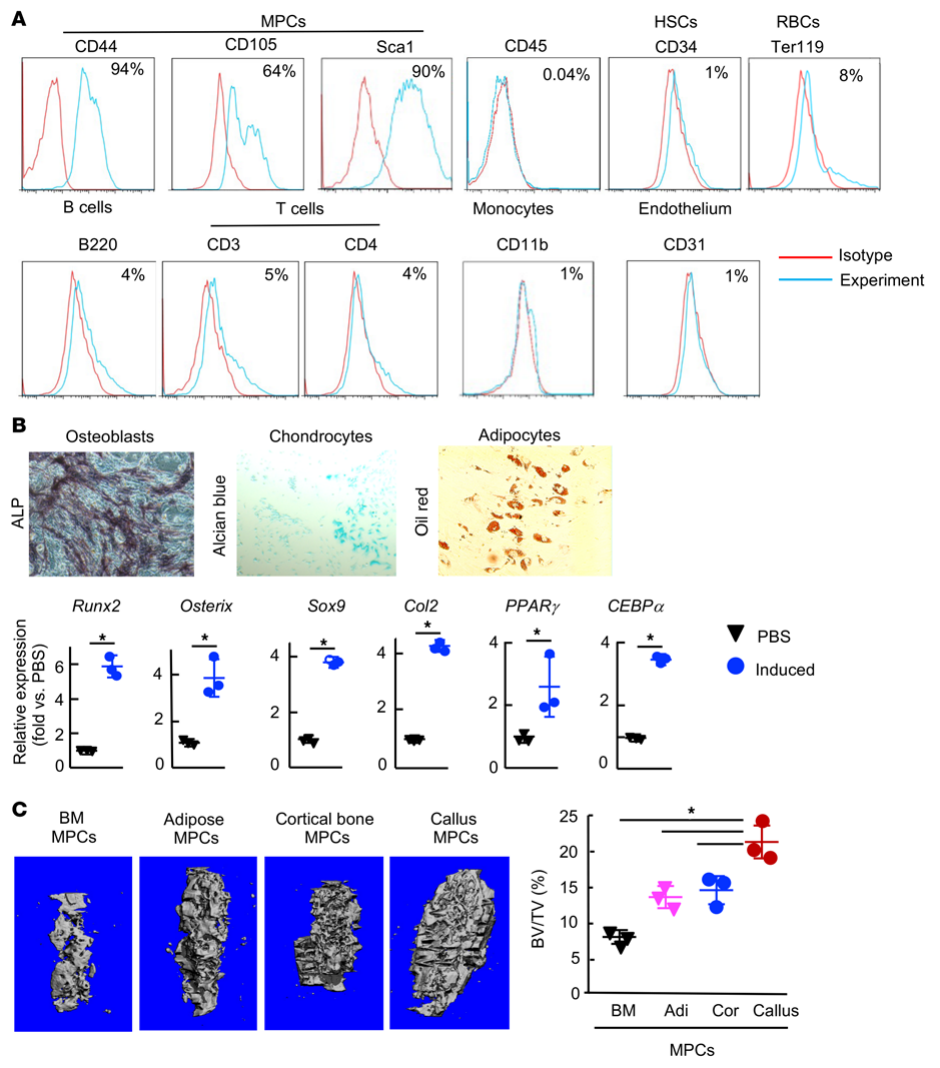
Callus-derived cells have properties of MPCs. Callus cells were isolated from young mice at 10 dpf. Third-passage cells were used. (**A**) Immunophenotypic analysis by flow cytometry showing cells expressing MPC surface markers. Data are representative of cells from 3 mice. (**B**) qPCR was performed to measure the expression of genes associated with OB, chondrocyte, or adipocyte differentiation 48 hours after cells were cultured in the various inducing media. *n =* 3 wells from a pool of 3 mice. Relative mRNA expression is the fold change versus PBS-treated cells as 1. *P < 0.05, by unpaired, 2-tailed Student’s *t* test. Original magnification, ×100. (**C**) In vivo bone formation assay. Micro-CT images show newly formed bone in cortical bone defects 6 weeks after cell injection. Adi, adipocytes; Cor, cortical bone–derived cells. *n =* 3. *P < 0.05, for callus versus the other groups, by 1-way ANOVA followed by Tukey’s post hoc test. Only comparisons between callus versus other bones are shown.

To determine whether SCs in callus affect MPCs and therefore fracture healing, we examined the effect of SCs from aged callus on MPCs derived from fracture callus. We first established a protocol to culture primary MPCs from callus, based on a published protocol (37) with some modifications, which enabled us to perform mechanistic studies using cells from young and aged mouse callus. We found that callus cells expressed MPC markers and could differentiate into OBs, chondrocytes, and adipocytes in vitro (Figure 4, A and B). To further examine the capacity of callus cells to form bone, we loaded callus cells onto decalcified bovine bone scaffolds and implanted them into tibial defects in recipient mice for 6 weeks, and then examined the volume of newly formed bone by micro-CT. We found that callus cells had the highest capacity to form new bone in vivo compared with BM-, adipocyte-, and cortical bone–derived cells (Figure 4C). Thus, we named them callus-derived MPCs (CaMPCs).

**Figure 5:**
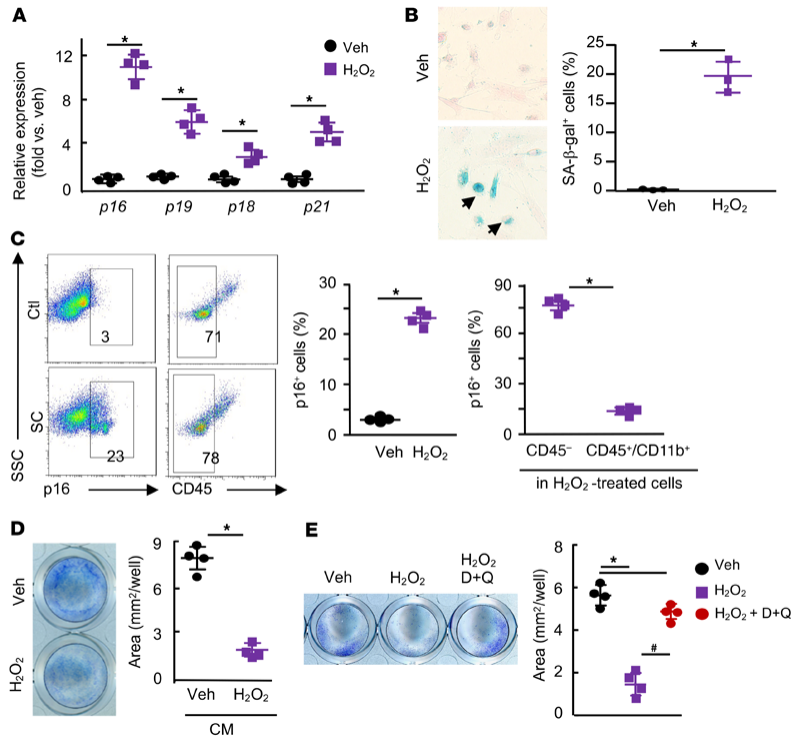
Callus cells treated with H_2_O _2_ inhibit the growth of CaMPCs, which is blocked by senolytic drugs. Callus tissues from young mice sacrificed at 10 dpf were used. (**A**) Callus pieces were treated with 50 μM H_2_O_2_ for 8 hours, changed to fresh culture media, and then cultured for an additional 2 days. Cells that grew out from and within callus pieces were harvested. Expression of *p16*, *p19*, *p18*, and *p21* was measured by qPCR. *n =* 4 wells. The experiment was repeated once. Relative mRNA expression is the fold change versus vehicle-treated cells as 1. (**B**) SA–β-gal staining was performed, and the percentage of SA–β-gal^+^ cells was measured by ImageJ. *n =* 3 wells. The experiment was repeated once. (**C**) The percentages of p16^+^ cells (left panel) and CD45^–^ and CD45^+^CD11b^+^ cells in p16^+^ cells (right panel) were determined by flow cytometry. *n =* 4 wells. The experiment was repeated once. (**D**) Callus pieces were treated with H_2_O_2_ as in **A** to generate SCs, and CM was collected. CaMPCs were treated with CM for 2 days. Cell growth assessed by methylene blue staining, and positively stained areas were measured by ImageJ. *n =* 4 wells. The experiment was repeated twice. *P < 0.05, by unpaired, 2-tailed Student’s *t* test (**A**–**D**). (**E**) Callus pieces were treated with H_2_O_2_ for 4 hours and then incubated with 200 nM dasatinib plus 20 μM quercetin or vehicle for 24 hours. CM was collected and used to treat CaMPCs for 2 days. Cell growth was assessed as described in **E**. *n =* 4 wells. *P < 0.05 vehicle versus other the groups; ^#^*P <* 0.05 H_2_O_2_ versus H_2_O_2_ plus D+Q, by 1-way anova followed by Tukey’s post hoc test. Veh, vehicle.

We also established a culture protocol, in which we generated callus SCs by treating bone and tissue pieces isolated from calluses of mice at 10 dpf with H_2_O_2_, a commonly used senescence inducer in vitro (38). We then collected conditioned medium (CM) from callus SCs, and treated CaMPCs with the CM. The rationale for developing this protocol is that cells within and attached to the callus pieces could become senescent in response to H_2_O_2_ treatment and release SASP factors into the CM, thus affecting CaMPCs. We found that cells from H_2_O_2_-treated callus pieces expressed high levels of senescence genes (*p16*, *p21*, *p18*, *p19*) (Figure 5A), and stained positively for SA–β-gal (Figure 5B) and p16 protein (Figure 5C). To further characterize these callus SCs, we used H_2_O_2_-treated cells from *p16^tdTom^* reporter mice, in which the *p16^tdTom^* reporter allele generates the fluorescent protein tdTomato under endogenous *p16^a^* regulation. This allele has been characterized extensively (39). We found that H_2_O_2_-treated cells were tdTomato^+^ and stained positively for SA–β-gal and γ-H2AX (Supplemental Figure 4). Using CD45 expression to separate CD45^+^ hematopoietic lineage from CD45^–^ mesenchymal lineage cells, flow cytometry revealed that 78% of callus SCs were CD45^–^ mesenchymal lineage cells (Figure 5C). We found that CM (Figure 5D) and cells that migrated from senescent callus pieces (data not shown) inhibited the growth of CaMPCs, which was prevented by the senolytic drugs D+Q (Figure 5E).

**Figure 6:**
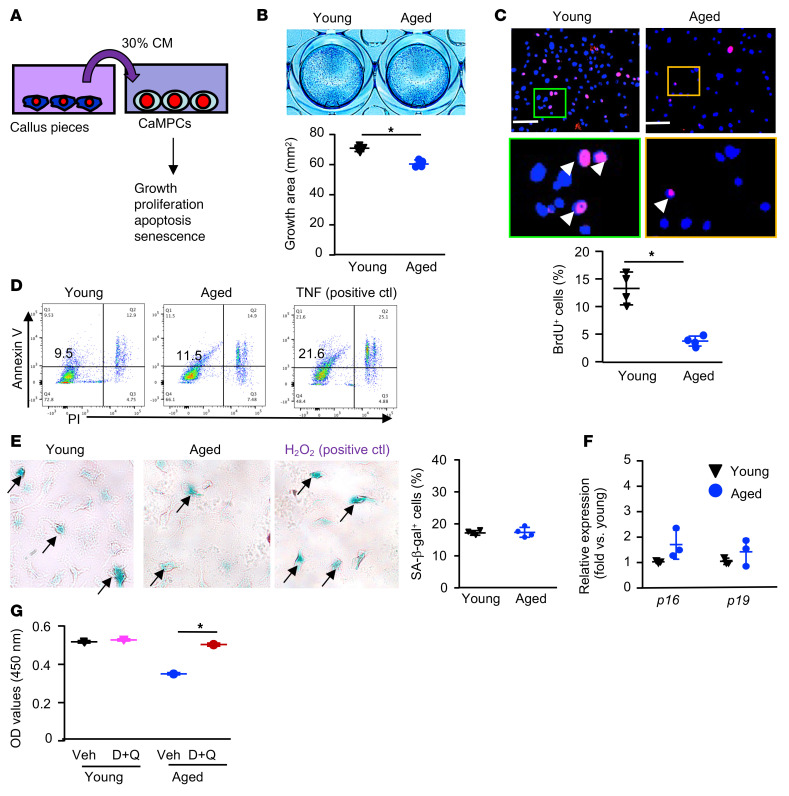
CM from aged callus inhibits the growth of MPCs, which is prevented by senolytic drugs. Young and aged mice were sacrificed on 10 dpf. (**A**) Callus pieces were cultured for 2 days to generate CM. CaMPCs were treated with 30% of CM for 2 days and subjected to growth, proliferation, apoptosis, and senescence analyses. (**B**) Cell growth, as in Figure 5E. *n =* 4 wells. The experiment was repeated twice. (**C**) Cell proliferation was assessed according to the percentage of cells incorporating BrdU. BrdU^+^ cells are indicated by white arrowheads. Scale bars: 100 mm. Original magnification, ×4 (enlarged insets). *n =* 4 wells. The experiment was repeated once. (**D**) Cell apoptosis was measured by flow cytometry as the percentage of annexin V^+^ cells. The experiment was repeated once. (**E**) Cellular was senescence assessed according to the percentage of SA–β-gal^+^ cells. *n =* 4 wells. The experiment was repeated once. (**F**) The expression of senescence markers was determined by qPCR. *n =* 3 wells. Relative mRNA expression is the fold change versus young cells as 1. The experiment was repeated once. *P < 0.05, by unpaired, 2-tailed Student’s *t* test (**B**, **C**, **E**, and **F)**. (**G**) CaMPCs were treated with CM with or without 200 nM dasatinib plus 20 μM quercetin. *n =* 4 wells. The experiment was repeated once. *P < 0.05, for vehicle versus D+Q, by 2-way ANOVA followed by Tukey’s post hoc test. Only comparisons between vehicle versus D+Q treatment in young or aged mice are shown. ctl, control.

Using this culture protocol, we examined the effects of CM from young and aged callus on the growth, proliferation, apoptosis, and cellular senescence of CaMPCs (Figure 6A). CM from aged, but not young, callus inhibited the growth and proliferation of CaMPCs (Figure 6, B and C), but had no effect on cell apoptosis (Figure 6D). We treated CaMPCs with young and aged callus CM or H_2_O_2_ as a positive control. We found that young and aged CM induced a comparable percentage of SA–β-gal^+^ cells and similar expression of senescence markers (Figure 6, E and F). Of note, H_2_O_2_ treatment induced increased SA–β-gal^+^ cells and senescence marker expression (data not shown). Similar to the observation that senolytic drugs blocked the inhibitory effect of H_2_O_2_-induced SCs on CaMPC growth (Figure 5G), D+Q also prevented the inhibitory effect of aged CM on CaMPC growth (Figure 6G).

### SCs in aged callus express high levels of TGF-β1, and TGF-β neutralization prevents the inhibitory effect of aged SCs on MPCs.

**Figure 7:**
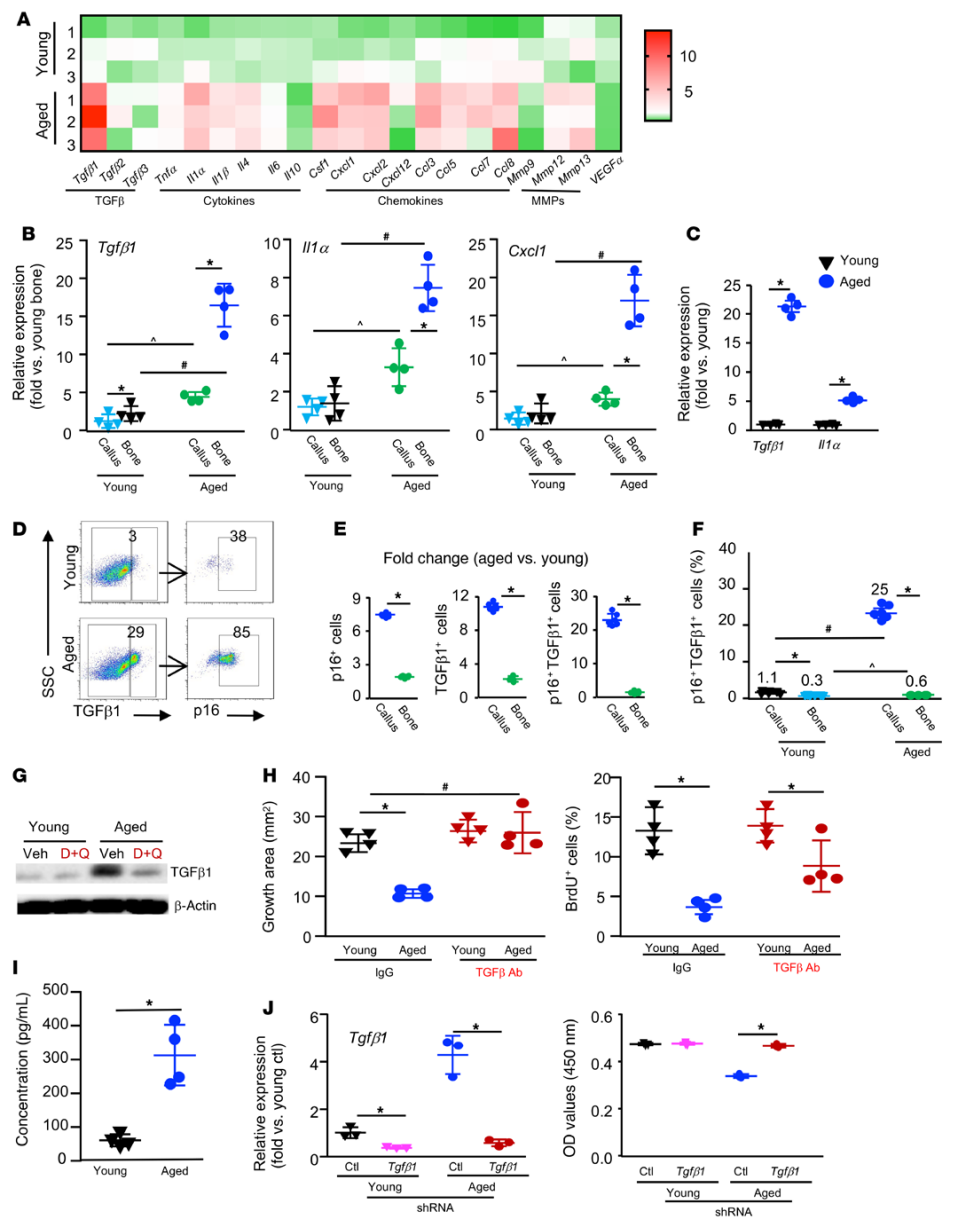
SCs in aged callus express high levels of TGF-β1, and TGF-β neutralization prevents the inhibitory effects of aged SCs on MPCs. Young and aged mice were sacrificed on 10 dpf. (**A**) Heatmap of the expression of SASP factors in callus as determined by qPCR. *n =* 3. (**B**) Expression of SASP factors in callus and nonfractured bone was assessed by qPCR. *n =* 4. Two-way ANOVA followed by Tukey’s post hoc test. (**C**) p16^+^ SCs isolated from young and aged callus. Expression of SASP factors examined by qPCR. *P < 0.05, by unpaired, 2-tailed Student’s *t* test. (**D**) p16^+^, TGF-β1^+^, and p16^+^TGF-β1^+^ cells were identified by flow cytometry. (**E**) Fold changes in p16^+^, TGF-β1^+^, and p16^+^TGF-β1^+^ cell percentages in callus and nonfractured tibiae of aged versus young mice. *n =* 4–6. *P < 0.05, for callus versus bone, by unpaired, 2-tailed Student’s *t* test. (**F**) Percentage of p16^+^TGF-β1^+^ cells in callus and nonfractured tibiae. *P < 0.05; ^*P <* 0.05; ^#^*P <* 0.05, for aged versus young, by 2-way ANOVA followed by Tukey’s post hoc test. (**G**) Expression of TGF-β1 in callus tissues following D+Q treatment by as determined by Western blotting. (**H**) CaMPCs were treated for 2 days with CM from young or aged callus with or without TGF-β–neutralizing Ab or IgG. Cell growth and proliferation were assessed by methylene blue staining or a BrdU incorporation assay. *n =* 4 wells. *P < 0.05, for IgG versus anti–TGF-β Ab; ^#^*P <* 0.05, for aged anti–TGF-β Ab versus young IgG, by 2-way ANOVA followed by Tukey’s post hoc test. (**I**) Concentration of active TGF-β1 in CM from young and aged callus cultures measured by ELISA. *P < 0.05, by unpaired, 2-tailed Student’s *t* test. (**J**) Callus pieces were harvested from young and aged mice and infected with *Tgfb1* or scrambled (Ctl) shRNA lentivirus. CM was collected. The expression of *Tgfb1* was measured by qPCR. CaMPCs were treated with the CM. Cell growth was assessed using a CCK8 kit. *n =* 3 wells. *P < 0.05, for control versus *Tgfb1* shRNA, by 2-way ANOVA followed by Tukey’s post hoc test. The experiment was repeated once.

One of the important features of SCs is SASP, which regulates cell function by a paracrine mechanism (40). To identify potential SASP factors that are expressed by aged callus SCs, we examined the expression profile of SASP factors in callus tissues from young and aged mice by quantitative PCR (qPCR). Among 21 SASP factors, 11 , including *Tgfb1* and *Il1a*, were expressed at a higher level in the aged callus than in the young callus. Of note, *Tgfb1*, but not *Tgfb2* or *Tgfb3,* was the most highly expressed SASP factor in aged callus (Figure 7A and Supplemental Figure 5). We also observed differences in mRNA expression levels of SASPs (*Tgfb1*, *Il1a*, and *Cxcl1*) between the fracture callus and contralateral nonfractured bone in both the young and aged groups, separately. We found that in young mice, the expression levels of *Tgfb1* were slightly, but significantly, higher in callus than in nonfractured bone and that the expression levels of other SASP factors were similar between callus and nonfractured bone. In contrast, in aged mice, the expression levels of all 3 SASP factors were markedly higher in callus than in nonfractured bone. The expression levels of these factors were higher in aged callus or nonfractured bone compared with levels in young mice (Figure 7B). We isolated SCs from young and aged callus using indirect magnetic labeling, in which SCs were sorted using an anti-p16 Ab (Supplemental Figure 6) and found that the expression of *Tgfb1* and *Il1a* in these SCs was increased 21- and 6-fold, respectively (Figure 7C). The percentage of p16^+^ and TGF-β1^+^ cells was significantly higher in callus from aged mice than in callus from young mice (Figure 7, D and E). Samples of nonfractured tibia from aged mice had approximately 2-fold more p16^+^, TGF-β1^+^, and p16^+^TGF-β1^+^ cells than did samples from young mice, and, more important, we observed a 25-fold increase in the number of p16^+^TGF-β1^+^ cells in callus over those in the contralateral nonfractured tibial bone from aged mice than in callus from young mice (Figure 7, E and F). The level of TGF-β1 in callus tissue was lower in samples from mice treated with D+Q than in samples from control mice (Figure 7G). To determine whether high mRNA levels of *Tgfb1* in aged-callus SCs would result in higher levels of biologically active TGF-β1, we treated CaMPCs with CM from aged callus in the presence of 1D11, a murine IgG1 mAb that neutralizes all 3 TGF-β isoforms of multiple species, including humans and mice (41). We found that 1D11 had no effect on the growth or proliferation of CaMPCs that were treated with young CM, but prevented the reduction in cell growth and proliferation induced by aged CM (Figure 7H). Of note, 1D11 also blocked the inhibitory effect of H_2_O_2_-induced SCs on the growth of CaMPCs (Supplemental Figure 7). High expression levels of active TGF-β1 in CM from aged-callus SCs were confirmed by ELISA (Figure 7I). To further examine the critical role of TGF-β1 in mediating SC-induced inhibition of CaMPCs, we used a shRNA approach to knockdown *Tgfb1* in callus tissue from which we collected CM. We treated CaMPCs with CM from *Tgfb1* shRNA– or control shRNA–infected callus pieces and found that the expression level of *Tgfb1* mRNA was decreased by 90% in *Tgfb1* shRNA–infected callus. Accordingly, CM from control shRNA–infected callus inhibited the growth of CaMPCs, which was abolished in CM from *Tgfb1* shRNA–infected callus, indicating that the inhibitory effect of aged callus CM was mediated by TGF-β1 (Figure 7J).

### Anti–TGF-β–neutralizing Abs enhance fracture healing in aged mice, which is accompanied by increased callus MPCs and cell proliferation.

**Figure 8:**
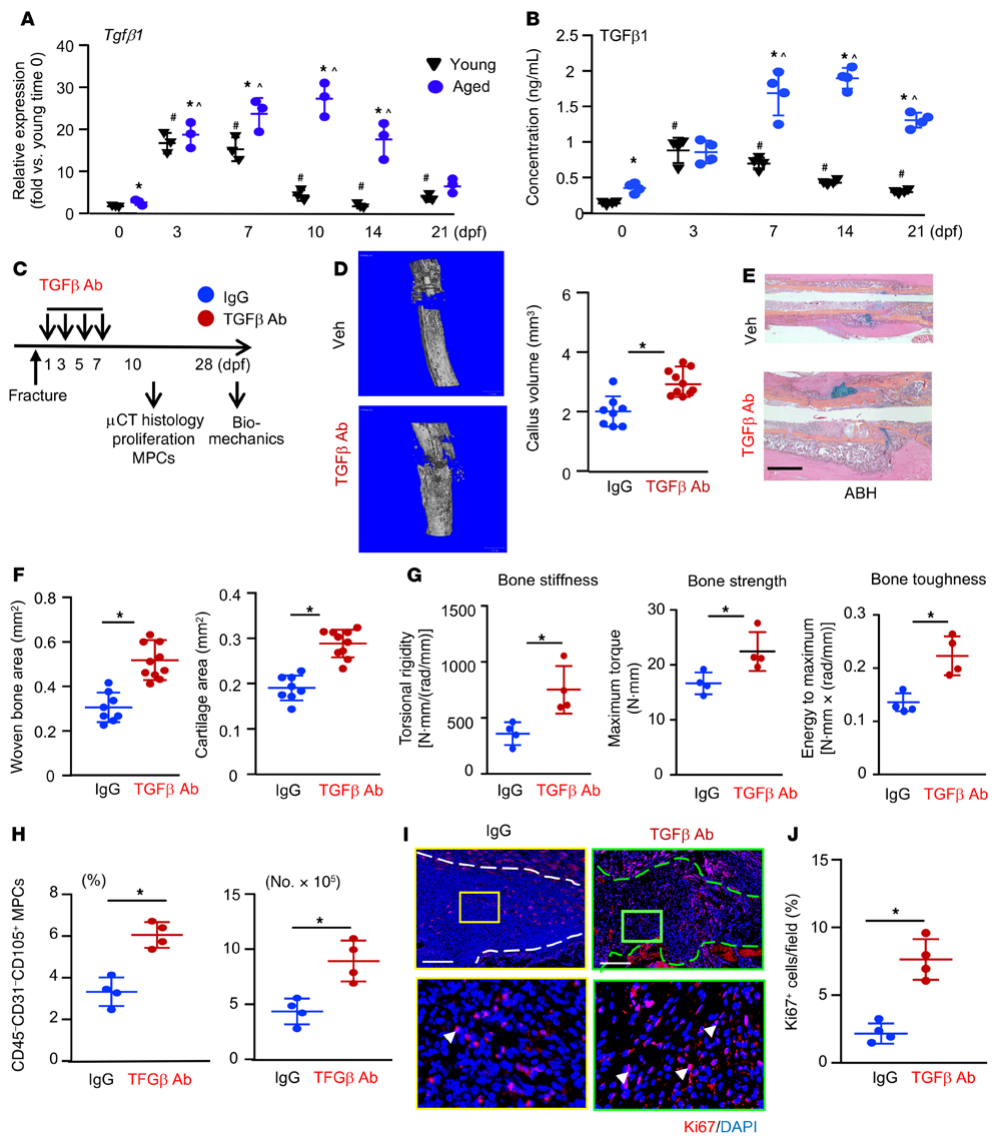
TGF-β neutralization enhances fracture healing in aged mice. Young and aged mice underwent tibial fracture surgery. (**A**) The expression of *Tgfb1* in fracture callus at indicated time points was measured by qPCR. *n =* 3. Relative mRNA expression is the fold-change versus young mice as 1. (**B**) The concentration of active TGF-β1 protein in fracture callus at indicated time points was measured by ELISA. *n =* 4. *P < 0.05, for aged versus young; ^#^*P <* 0.05, for young versus young 0 dpf; ^*P <* 0.05, for aged versus aged 0 dpf, by 2-way ANOVA followed by Tukey’s post hoc test (**A** and **B**). (**C**) Outline of the experimental design. Aged mice were given 2 μg in 10 μL TGF-β Ab, 1D11, or isotype IgG vehicle by intra-callus injection on 1, 3, 5, and 7 dpf and sacrificed on 10 dpf (**D**–**F** and **H**–**J**) or 28 dpf (**G**). *n =* 4–5. (**D**) Callus volume was measured by micro-CT. *P < 0.05, by unpaired, 2-tailed Student’s *t* test. (**E**) Representative images of ABH-stained sections showing more woven bone and callus areas in the anti–TGF-β Ab–treated mice. Scale bar: 1 mm. (**F**) Woven bone and cartilage areas were analyzed using Visiopharm software. (**G**) Bone stiffness, strength and toughness were assessed by biomechanical testing at 28 dpf. (**H**) The percentage and number of MPCs identified as CD45^–^CD31^–^CD105^+^ cells in fracture callus were determined by flow cytometry. (**I**) Representative paraffin sections of callus immunostained with anti-Ki67 Ab to detect proliferating cells (arrowheads). External callus is indicated by the dashed lines. Scale bars: 500 μm. Original magnification, ×4 (enlarged insets). (**J**) The percentage of Ki67^+^ cells was quantified by ImageJ. *P < 0.05, by unpaired, 2-tailed Student’s *t* test (**F**, **G**, **H**, and **J**).

The role of TGF-β in bone healing is complicated. An early study reported that a single local injection of TGF-β1 into bone defects enhanced healing in 22-month-old rats (42). A recent study reported that mice with constitutively active TGF-β1 in OBs had generalized bone loss that was restored by short-term treatment with a bone-targeted TβR1 inhibitor (17). We reported that aged mice and elderly humans had increased levels of active TGF-β1 in their bone and BM and that TGF-β1 inhibited OB differentiation from MPCs (18). On the basis of these findings and the rescuing effect of anti–TGF-β–neutralizing Ab inhibition of CM-mediated CaMPCs in aged callus (Figure 7H), we hypothesized that SCs in callus from aged mice produce excessive TGF-β1, which would inhibit the proliferation of callus MPCs and delay fracture healing, and that this can be prevented by TGF-β neutralization. The expression levels of *Tgfb1* mRNA and protein in callus from young and aged mice at different time points after fracture surgery were measured by qPCR (Figure 8A) and ELISA (Figure 8B), respectively. Nonfractured bone samples (time 0) were included as controls. Nonfractured bone samples from aged mice had slightly, but significantly, increased TGF-β1 expression at both mRNA and protein levels than did those from young mice. In fracture callus from young mice, *Tgfb1* mRNA levels were markedly increased, peaked at the early phase (3 and 7 dpf) of fracture repair, declined at 10 dpf, and returned to nonfractured bone control levels at later phases (14 and 21 dpf) (Figure 8A). The expression profile of TGF-β1 protein was similar to that of mRNA, but to a lesser extent (Figure 8B). Interestingly and importantly, we found that TGF-β1 levels were higher in callus samples from aged mice than in those from young mice, which persisted until the later phase of the fracture repair process (Figure 8, A and B). The persistently high expression level of TGF-β1 was more obvious in the active protein form of TGF-β1 than *TGFB1* mRNA. We also measured the concentration of active TGF-β1 in peripheral blood of young and aged mice at the same time points following fracture that we used in Figure 8, A and B. We found that, similar to callus local TGF-β1 levels, serum TGF-β1 levels were also increased in aged mice (Supplemental Figure 8A).

To determine whether TGF-β blockage could promote fracture healing in aged mice, we injected 1D11 or isotype IgG into the callus of aged mice at 1, 3, 5, and 7 dpf — a protocol that we used for the delivery of senolytic drugs (Figure 2)— and assessed fracture healing by micro-CT, histomorphometry, and biomechanical testing, as well as callus MPC numbers and cell proliferation (Figure 8C). The rationale for using aged mice only was that, (a) unlike the elderly, in which enhanced fracture healing could reduce fracture-related complications, fracture healing in healthy young individuals occurs rapidly and does not need medication; and (b) callus TGF-β1 levels were persistently higher in aged mice than young mice (Figure 8, A and B), which likely plays a detrimental role in fracture healing. Mice that were treated with 1D11 had significantly higher callus volume (micro-CT, Figure 8D), cartilage and new bone area (Figure 8, E and F), and bone strength (Figure 8G) than did IgG-treated mice. Furthermore, TGF-β neutralization significantly increased the percentage and number of CD45^–^CD31^–^CD105^+^ MPCs (Figure 8H and Supplemental Figure 8B) and Ki67^+^ proliferating cells in callus (Figure 8, I and J).

### Senolytic drugs enhance fracture healing in aged female mice and reduce the expression of SASP factors in callus tissues.

**Figure 9:**
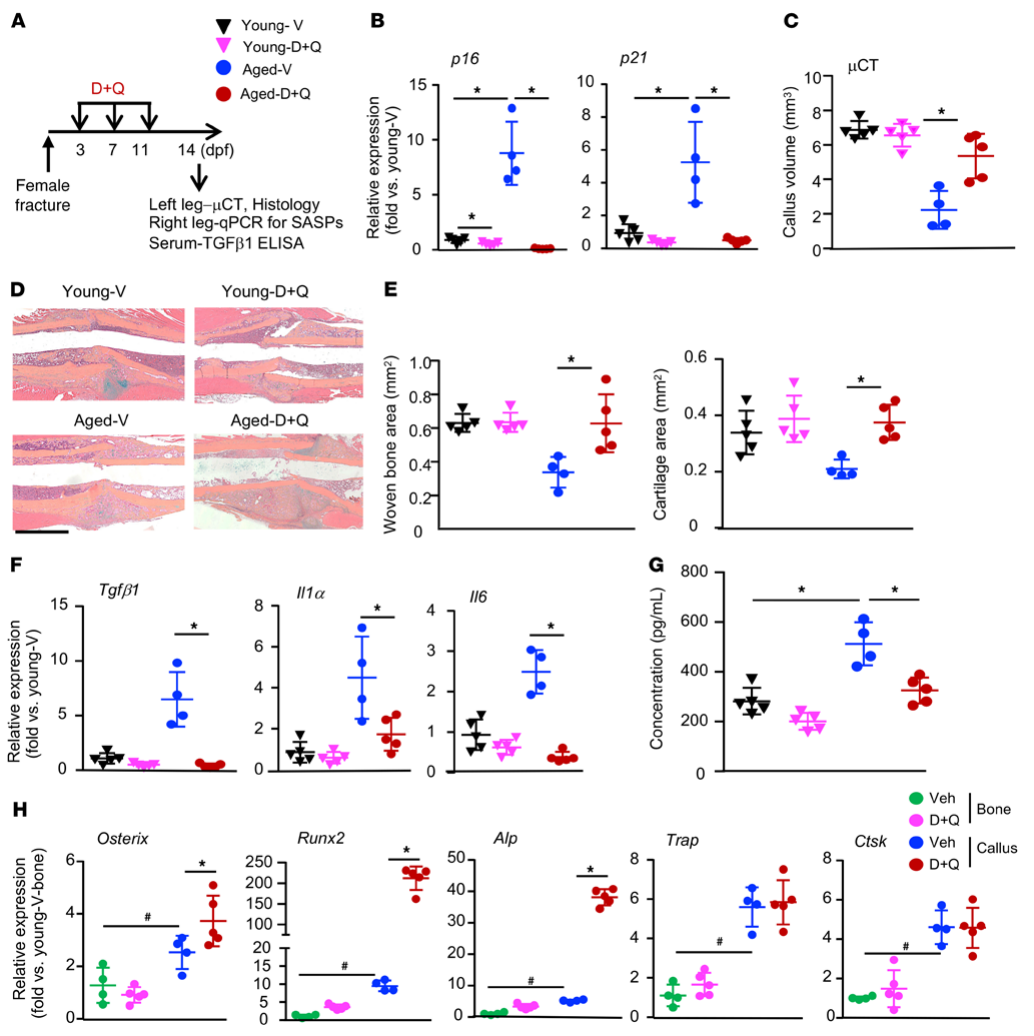
Senolytic drugs enhance fracture healing in female aged mice and reduce the expression of SASP factors in callus tissues. Young and aged female mice underwent tibial fracture surgery. (**A**) Experimental outline. Mice were given 5 mg/kg dasatinib plus 50 mg/kg quercetin or vehicle by gavage on 3, 7, and 11 dpf and sacrificed on 14 dpf. *n =* 4–5. (**B**) The expression of *p16* and *p21* in callus tissues was determined by qPCR. Relative mRNA expression is the fold change versus vehicle-treated young mice as 1. (**C**) Callus volume was measured by micro-CT. (**D**) Representative images of ABH-stained sections showing more woven bone and callus areas in D+Q-treated aged mice than in vehicle-treated aged mice. Scale bar: 1 mm. (**E**) Woven bone and cartilage areas were analyzed using Visiopharm software. (**F**) The expression of SASP factors in callus tissues was measured by qPCR. Relative mRNA expression is the fold change versus vehicle-treated young mice as 1. (**G**) The concentration of active TGF-β1 protein in serum was measured by ELISA. *P < 0.05, for vehicle versus D+Q or vehicle young versus vehicle aged, by 2-way ANOVA followed by Tukey’s post hoc test (**B**, **C**, and **E**–**G**). (**H**) The expression of OB and OC marker genes in nonfractured bone and callus was determined by qPCR. Relative mRNA expression is the fold change versus bone samples from vehicle-treated young mice as 1. *P < 0.05 vehicle versus D+Q; ^#^*P <* 0.05, for vehicle young bone versus vehicle aged bone, by 2-way ANOVA followed by Tukey’s post hoc test.

To determine whether senolytic drugs can promote fracture healing in female mice, we repeated the D+Q experiment in young and aged female mice and assessed the expression of senescence markers (*p16*, *p21*) and SASP factors (*Tgfb1*, *Il1a*, *Il6*) (Figure 9A). In these experiments, we treated mice at 3, 7, and 11 dpf, time points at which the increase in SCs occurs and during the period of peak increased SC numbers (day 10) in the callus of aged mice, as shown in Figure 1A. We found that, compared with young mice, aged mice had significantly increased expression of the senescence markers *p16* and *p21*, and this was prevented by the D+Q treatment (Figure 9B). D+Q had minor (*p16*) or no (*p21*) effect on the expression of senescence markers in young mice (Figure 9B). As shown in male mice in Figure 3, D+Q increased callus volume (Figure 9C) and the woven bone/cartilage area (Figure 9, D and E) in aged, but not young, mice. Accordingly, D+Q also decreased the expression levels of the SASP factors *Tgfb1*, *Il1a*, and *Il6* in callus of aged mice (Figure 9F). To determine whether fracture increases systemic TGF-β1 levels in aged mice and if this can be blocked by SC clearance, we measured active TGF-β1 concentrations in serum by ELISA. We found that aged mice had significantly higher levels of serum TGF-β1 following fracture than did young mice and that this was prevented by D+Q treatment (Figure 9G).

To examine whether D+Q has off-target effects, we measured the expression levels of genes related to OBs and osteoclasts (OCs) in fracture callus and nonfractured long bones from aged mice. We anticipated that D+Q treatment would not affect OB or OC gene expression in long bone samples, because there are few SCs in long bones. However, we hypothesized that D+Q might affect OBs in callus samples, because D+Q can attenuate the inhibitory effect of SCs on OBs by removing SCs. We found that D+Q had no effect on OB genes in nonfractured bones but that this treatment significantly increased OB marker gene expression, indicating that our D+Q regimen likely had no off-target effects on OBs (Figure 9H). Finally, to determine whether D+Q treatment has adverse effects, we examined internal organs including the brain, lungs, heart, liver, spleen, kidneys, and gastrointestinal (GI) tract and did not observe obvious abnormalities, such as tumors or bleeding. Body weights were similar for the D+Q- and vehicle-treated mice (Supplemental Figure 9).

## Discussion

Cellular senescence is clearly emerging as a main player in aging and age-associated diseases (43, 44). Several clinical trials of senolytic drugs that kill SCs obtained encouraging results (34, 45, 46). In the current study, we extended the use of senolytic drugs to aging fractures and demonstrated that short-term administration of the senolytic drugs dasatinib and quercetin promoted fracture healing in aged mice. SCs affect cell function by releasing SASP factors. We found that SCs from the fracture callus of aged mice produced a large amount of TGF-β1 that inhibited the growth of CaMPCs. Intra-callus injection of anti–TGF-β–neutralizing Abs enhanced fracture healing in aged mice, which was accompanied by increased callus MPCs. We proposed a working model in which fracture triggers increased cellular senescence in the callus of aged mice, which would inhibit MPCs by expressing TGF-β1. In our model, this inhibition could be prevented by senolytic drugs or TGF-β neutralization.

Cellular senescence is trigged by many mechanisms, including aging, genomic or epigenomic damage, and tissue injury (47). In young mice, acute injury also causes cellular senescence. For example, anterior cruciate ligament transection (ACLT) induces posttraumatic OA in 10-week-old mice and increases the number of senescent chondrocytes (24). Interestingly, we found that the number of SCs was markedly increased in the fracture callus of aged mice compared with SCs observed in nonfractured sites of aged mice and fracture callus of young mice (Figures 1 and 2), suggesting that combined acute injury and natural aging could robustly trigger cellular senescence. Thus, unlike treating osteoporosis induced by natural aging, in which senolytic therapy is given infrequently (e.g., once a month) over a long period of time (23), aged fractures required short-term administration of senolytic drugs repeatedly and frequently to deplete the rapidly accumulated SCs (Figure 3).

We found that D+Q treatment promoted fracture healing in aged mice. The effect on young mice was not observed with the present treatment regimen (Figures 3 and 9). This differs from the study of posttraumatic OA, in which the senolytic drug UBX0101 effectively reduced ACLT-induced joint tissue damage in both young (10-week-old) and aged (20-month-old) mice (24). Currently, we do not know why our senolytic treatment was effective only in aged mice. This may be due to consistently higher levels of TGF-β1 derived from SCs in aged mice than in SCs from young mice, leading to MPC growth inhibition (Figure 8, A and B). Another possibility is that SCs in the fracture callus (long bone) may be different from the SCs in ACLT-induced OA (joint), resulting in different responses to D+Q and UBX0101. Cellular senescence occurs in multiple cell types, including bone cells. In our preliminary study, callus SCs were composed of cells from both mesenchymal and myeloid lineages (Supplemental Figure 10), while in ACLT-induced OA, the majority of SCs consist of chondrocytes. It will be interesting to compare the transcriptome in callus SCs in young and aged mice to determine whether they express different genes or SASP factors.

Treatment with D+Q has been reported to improve pathological conditions in both preclinical and clinical settings, including for age-associated osteoporosis and OA (48). However, these studies used long-term administration, in which D+Q was given for months, with an aim to eliminate SCs that accumulate during the aging or disease processes. Recently, Khosla et al. reported that D+Q enhanced fracture healing in young adult mice with an intermittent treatment regimen during the whole healing process (27). Potential side effects of D+Q due to long-term administration may limit their clinical use for patients with nonlethal or nonsevere diseases. In contrast, we used a short-term protocol, with a total of 3 doses, as shown in Figure 9. Our study indicates that under certain aging conditions in which additional acute injury causes a rapid increase in SCs locally, short-term D+Q treatment may have beneficial effects. We are investigating whether other senolytic drugs may have a similar beneficial effect on fracture healing in aging mice.

Dasatinib affects bone remodeling via the regulation of both OBs and OCs (49, 50). When given twice a day for 3 or 7 weeks, dasatinib promoted fracture healing in young mice by increasing OB differentiation (49). Thus, it is possible that D+Q improved fracture healing through off-target effects on non-SCs such as OBs. However, we think that this is unlikely, because we administered D+Q only 3 times, which had no effect on fracture healing in the young mice. Further, D+Q had no effect on the expression levels of OB marker genes in nonfractured long bones (Figure 9H), supporting the notion that D+Q work through clearance of SCs, because young mice have far fewer SCs than do aged mice.

NSAIDs are widely used in the treatment of fracture pain in humans (51). However, whether NSAIDs promote fracture healing is controversial. Early studies reported that NSAIDs impair bone healing in mice (52, 53) because of the potential anabolic role of inflammatory cells through released inflammatory cytokines (54). However, Josephson et al. (7) recently reported that NSAID treatment enhances fracture repair in middle-aged mice, possibly due to modulation of increased inflammation in these animals. We have consulted with our orthopedic colleagues about the use NSAIDs in their elderly patients with fractures to promote fracture healing. They reported that they are unwilling to do so, because it is very difficult to control when the NSAIDs are given, and periods of acute inflammation are beneficial for fracture healing. Additionally, several clinical trials have reported that NSAIDs carry the risk of ectopic bone formation as well as nonunion and delayed union (55, 56). Given all of this information, we concluded that NSAID treatment for fracture healing is complex, prior results are variable and hard to clearly explain, and that it would not serve as a good control in our study.

A recent study reported increased mesenchymal cell senescence in aged mice with bone fractures and that inhibiting NF-κB activation not only enhanced fracture healing but also decreased SC numbers (7). However, this study used cells derived from BM and did not examine whether SCs affected MPCs. We used callus MPCs as our cell model by directly deriving them from primary callus tissue. We believe that CaMPCs have several advantages over BM-derived MPCs for the study of fracture healing, because they come from a unique microenvironment where they are exposed to factors that are present only in fracture sites. Indeed, our in vivo bone formation assay revealed that CaMPCs have higher bone-forming capacity than do BM-derived MPCs (Figure 4). The bone-forming capacity of CaMPCs is similar to that of MPCs derived from periosteum. This is not surprising, because the majority of callus MPCs are from the periosteum (57). We do not know the origin of these CaMPCs, which we plan to study using single transcriptome analysis.

SCs produce the SASP; therefore, they affect target cells in a variety of ways. We found that aged callus SCs inhibited the proliferation and growth of CaMPCs, but not their apoptosis or senescence (Figure 6). This is very important, because the growth and expansion of MPCs play a critical role in the early phase of fracture healing (58). MPCs migrate from periosteum and, to a lesser degree, extend from BM to the fracture site and expand and differentiate into chondrocytes, OBs, and pericytes. All of these cell types are required for callus and bone formation and angiogenesis. SCs would affect all these cellular processes.

Among 21 SASP factors (10), we found that *Tgfb1* was the most highly expressed in aged callus (Figure 7). Early studies demonstrated that TGF-β1 or SMAD3 promotes fracture healing (59). However, we and others recently reported that high TGF-β1 levels in aging mice inhibit OB differentiation (18, 59). Thus, the role of TGF-β1 in fracture healing is complicated and needs to be investigated further. There is a general notion that TGF-β1 is released mainly from bone matrix as a result of OC-mediated bone resorption to affect age-associated bone loss (18, 60). However, bone remodeling occurred in the late phase of fracture healing, while TGF-β blockade, given 1 day after fracture, promoted fracture healing and increased callus MPCs (Figure 8). Furthermore, callus SCs expressed high levels of TGF-β1 (Figure 7). These findings raise 2 key points: (a) TGF-β1 in aged bone comes not only from bone matrix, but also from cells such as SCs; (b) targeting TGF-β1 at the early phase of fracture healing might have beneficial effects in the elderly. Our data (Figure 7H) demonstrate that the TGF-β Ab had only a partial effect on cell proliferation, as assessed by a BrdU incorporation assay. One explanation is that BrdU marks only proliferating cells and that during the early phase of fracture repair, cells participating in the cell cycle constitute only a portion of the proliferating cells. Another possibility is that SASP factors, other than TGF-β1, may contribute to the inhibitory effects, which we plan to investigate in future studies.

We found much higher *Tgfb1* mRNA expression levels in purified SCs from aged as opposed to young callus (Figure 7C). Early studies reported that *TGFB1* transcription is upregulated by multiple factors, including retinoblastoma (RB) (61), a key factor in the classic senescence pathway. The TGF-β1 promoter region contains potential binding sequences for NF-1, AP-1, and SP1 (62–64). SP1, a member of the specificity protein/Kruppel-like factor family, promotes the transcription of p16 (65) and dephosphorylates RB (66), generating an active, hypophosphorylated form of RB in the classic senescence pathway. Therefore, TGF-β1 expression is closely associated with cellular senescence. It will be interesting to determine how TGF-β1 is upregulated in these aged SCs.

There are several limitations of the current study. First, we do not know the specific cell types of callus SCs and their association with other cell types. We also do not know if callus SCs from aged mice represent a distinguishing SC subset that differs from callus SCs of young mice or bone/BM SCs of aged mice. Our original plan to answer these questions was to perform single-cell RNA-Seq using purified SCs from the callus of young versus aged mice and SCs from BM of aged mice. However, we faced several technical difficulties when trying to isolate high-quality RNA from SCs. Using *p16-3MR* mice (25), we were unable to detect transgene-expressing cells in fracture callus. Second, we have not optimized the senolytic therapy regimen, since we used only short-term protocols for senolytic drug treatment (Figures 3 and 9). Third, there remains a crucial need to identify factors other than TGF-β1, because TGF-β neutralization only partially rescued the inhibitory effects of callus SCs on MPCs. Finally, we used CaMPCs from young mice, because our focus was on testing the effect of young and aged callus SCs on MPCs. However, it will be interesting to determine if aged CaMPCs respond to SCs differently from young cells.

In summary, using primary SCs and MPCs derived from fracture callus, we found that aged mice had a significantly higher number of SCs in callus than did young mice or in nonfractured bones of aged mice. The senolytic drugs D+Q, given 1 dpf for a short period, enhanced fracture healing in the aged mice. Aged callus SCs inhibited the growth and proliferation of CaMPCs and expressed high levels of TGF-β1. An anti–TGF-β–neutralizing Abs prevented the inhibitory effects of aged SCs on MPCs and promoted fracture healing in aged mice. Thus, fracture injury triggers significant senescence of cells in the callus tissue of aged mice, leading to increased expression of TGF-β1 and inhibition of MPC growth and fracture repair. Senolytic drugs represent a promising therapy for enhancing fracture repair in the elderly by eliminating SCs, which delay fracture repair by expressing TGF-β1, which we have identified as a molecular inhibitor of repair.

## Methods

### Mice.

Mice were housed in the vivarium under specific pathogen–free conditions. Young (4 months old, equivalent to a 26-year-old human) and aged (20 months old, equivalent to a 60-year-old human) male and female C57BL/6J mice from the National Institute on Aging, NIH, were used. Mice were randomized and grouped according to body weight. *p16^tdtomato^* reporter mice, originally generated in the Sharpless laboratory (39), were used in the supplemental experiments.

### Tibial fracture model and animal treatments.

The open tibial fracture procedure is a standard operating procedure (SOP) used in the UR Center for Musculoskeletal Research (CMSR) (36, 67, 68). Briefly, a 5 mm long incision was made in the skin over the anterior tibia after administration of anesthesia. A sterile 27 gauge needle was inserted via the proximal tibial articular surface into the BM cavity, temporarily withdrawn to facilitate tibial midshaft transection using a scalpel, and reinserted to stabilize the fracture, which was confirmed by x-ray. The incision was closed with sutures. Mice received 0.5 mg/kg buprenorphine SR s.c. to control pain. To study the effect of SC depletion, we used the published senolytic drugs dasatinib (5 mg/kg, MilliporeSigma, catalog CDS023389) and quercetin (50 mg/kg, MilliporeSigma, catalog PHR1488) (23, 69, 70). Mouse tibiae were fractured and given D+Q or vehicle by gavage at 1, 3, 5, and 7 dpf for male mice and 3, 7, and 11 dpf for female mice. To study the effect of TGF-β inhibition, the anti–TGF-β–neutralizing Ab 1D11 (2 μg/10 μL, R&D, catalog/clone MAB1835/1D11) or vehicle (mouse IgG1 isotype control, R&D Systems, catalog/clone MAB002/11711) was injected into the callus at 1, 3, 5, and 7 dpf, the same time points as for administration of the senolytic drugs. 1D11 is a mAb against all 3 TGF-β isoforms (TGF-β1, -2, -3) that has been used in clinical trials (71).

### Preparation of callus-derived MPCs and cell multiple-lineage differentiation assay.

Mice were euthanized by CO_2_ inhalation and secondary cervical dislocation for tissue harvesting at 10 dpf. The callus was dissected free of soft tissue and cut into pieces. Callus pieces were washed thoroughly with cold PBS and then digested with ACCUMAX cell detachment solution (STEMCELL Technologies) for 30 minutes at room temperature, followed by culturing in basal medium (α-MEM containing 15% FBS). Cells that migrated from callus pieces were cultured in the basal medium to confluence and identified as CaMPCs. CaMPCs from passages 3–5 were used for the experiments. Callus cells (8 × 10^5^ to 1.5 × 10^6^) were obtained from each mouse (2 fractures). For OB differentiation, CaMPCs were cultured for 3 days in α-MEM culture medium containing 10% FBS with 50 μg/mL ascorbic acid and 10 mM β-glycerophosphate. For adipocyte differentiation, CaMPCs were cultured for 5 days in α-MEM culture medium containing 10% FCS, 10 nM dexamethasone, 5 μg/mL insulin, 100 nM indomethacin, and 0.5 mM methylisobutylxanthine (all from MilliporeSigma). For chondrocyte differentiation, CaMPCs were seeded for micromass culturing (1 × 10^5^ cells in 10 μL) for 1.5 hours and then cultured in 40% DMEM/60% F12 with 10% FBS, 10 mM β-glycerophosphate, and 50 μg/mL ascorbate for 7 days. Cells were then stained for alkaline phosphatase (ALP) activity for OB, with Oil red for adipocyte identification and Alcian blue for chondrocyte identification.

### In vivo bone formation assay.

The bone-forming capacity of MPCs from different sources was measured using an in vivo bone defect model we described previously (72). In brief, 2 × 5 mm cortical bone defects were made in the anterior proximal tibiae of anesthetized 2-month-old SCID mice and filled with bovine bone matrix. MPCs (5 × 10^5^) derived from BM (72), cortical bone (72), adipose tissue (73), and fracture callus were injected into the bone matrix of the defects. The mice were sacrificed 6 weeks after surgery. The volume of new bone formed in the defects was measured by micro-CT.

### Generation of CM and cell phenotype analyses.

Mice were sacrificed at 10 dpf. Calluses were cut into pieces and cultured in αMEM medium containing 15% FBS for 2 days, and the CM was collected. For analysis of cell phenotype, CaMPCs were treated with 30% CM or PBS for 2 days and then subjected to 3 different assays. (a) Methylene blue staining was used to determine growth, in which the cells were fixed in 10% formalin and stained with 1% methylene blue solution. The percentage of positively stained area/well was calculated by ImageJ software (NIH) on scanned images. In some of the experiments, a cell counting kit 8 (CCK8) (Abcam, catalog ab228554) was used according to the manufacturer’s instructions. (b) Proliferation was assessed using a BrdU incorporation assay. Cells were cultured on chamber slides and incubated with BrdU (10 μM, Invitrogen, Thermo Fisher Scientific, catalog 000103) for 3 hours. The chamber slides were fixed in 10% formalin and stained with a rabbit anti-BrdU Ab (1:500, Abcam, catalog 152095), followed by a goat anti–rabbit Alexa Fluor 568 Ab (1:400, Invitrogen, Thermo Fisher Scientific, catalog A-11011), and then covered with Vectashield Mounting Medium with DAPI (Vector Laboratories, catalog H-1200) to visualize nuclei. The percentage of BrdU^+^ stained cells versus the total number of cells was quantified using ImageJ. (c) Apoptosis was assessed using an annexin V–FITC apoptosis kit (Thermo Fisher Scientific, catalog V13242), and propidium iodine (PI) (MilliporeSigma, catalog 81845) was used as a counterstain, as described by the manufacturer. Cells were stained with FITC annexin V and PI working solution and then analyzed using a BD LSR II flow cytometer. The percentage of annexin V–PI apoptotic cells was calculated. To examine cellular senescence, CaMPCs cultured on chamber slides were treated with 30% CM or PBS for 5 days and stained with SA–β-gal (Cell Signaling Technology, catalog 9860) following the manufacturers’ instructions. The percentage of SA–β-gal^+^ cells versus the total number of cells was calculated in 5 random high-power fields at ×200 magnification and expressed as the mean.

### Histology and histomorphometric analysis.

Tibiae were fixed in 10% formalin and decalcified in 10% EDTA. Paraffin sections were prepared for histology, and 4 μm thick sections were cut at 3 levels (each level was cut 50 μm apart). Sections were stained with Alcian blue/hematoxylin (ABH) and counterstained with eosin/Orange G, following by scanning with an Olympus VS-120 whole-slide imaging system. Scanned images were labeled by numbers, and histomorphometric measurement of cartilage and woven bone area was performed using Visiopharm software (version 2018.4) following CMSR SOPs in a blinded manner (74, 75).

### Biomechanical testing.

Fresh tibiae at 28 dpf were stored at –80°C after carefully removing the stabilizing needle to avoid any damage to the architecture of the callus. The tibial ends were embedded in polymethylmethacrylate and placed on an EnduraTec system (Bose). A rotation rate of 10/s was used to twist the samples to failure or up to 80°. Maximum torque, maximum rotation, and torsion rigidity were analyzed following CMSR SOPs (35).

### Immunostaining and data analysis.

Immunostaining was performed on paraffin or frozen sections. For p16 staining to identify SCs, tibiae were embedded in Tissue-Tek and sectioned into 8 μm pieces using a Leica CM1850 cryostat (Leica). Frozen sections were subjected to immunofluorescence staining with rabbit anti–mouse p16 Ab (1:100, Invitrogen, Thermo Fisher Scientific, catalog PA1-46220), followed by goat anti–rabbit Alexa Fluor 568 Ab (1:400, Invitrogen, Thermo Fisher Scientific, catalog A-11011), and then covered with Vectashield Mounting Medium with DAPI. The specificity of the p16 Ab was confirmed using *p16*-deficient cells (Supplemental Figure 2). For γ-H2AX staining to identify cells with DNA double-stranded breaks, frozen sections were incubated with unconjugated affinity-purified F(ab) fragment anti–mouse IgG (H+L) (1:400, Jackson ImmunoResearch, catalog 115-007-003) to block endogenous IgG. The sections were subjected to immunofluorescence staining with mouse anti–mouse γ-H2AX Ab (1:500, MilliporeSigma, catalog 05-636-I), followed by goat anti–mouse Alexa Fluor 488 Ab (1:400, Abcam, catalog ab150113), and then covered with Vectashield Mounting Medium with DAPI. For Ki67 staining to detect proliferating cells, paraffin sections (4 μm thick) were subjected to antigen retrieval in citrate buffer (10 mM citric acid in 0.05% Tween-20, pH 6.0) at 60°C overnight. The sections were stained with rabbit anti-Ki67 Ab (1:1000, Abcam, catalog ab15580), followed by goat anti–rabbit Alexa Fluor 568 Ab (1:400, Invitrogen, Thermo Fisher Scientific, catalog A-11011), and then covered with mounting medium with DAPI. For SA–β-gal staining to identify SCs, frozen sections were stained for SA–β-gal with a kit from Cell Signaling Technology (catalog 9860S) following the manufacturer’s instructions. SCs were identified as blue-stained cells. For data analysis, the stained sections were scanned with an Olympus VS-120 whole-slide imaging system. Scanned images were numbered, and the external callus area was outlined. The number of positively stained cells and total cells was quantified with ImageJ software. The percentage of positively stained cells versus total cells, or the number of positively stained cells within the external callus, was calculated.

### Flow cytometry and SC enrichment.

To identify cell surface markers, cells were stained with various fluorescein-labeled Abs, including anti-CD44, anti-CD105, anti-Sca1, anti-CD45, anti-CD34, anti-Ter119, anti-B220, anti-CD3, anti-CD11b, and anti-CD31 Abs (details on these Abs are listed in Supplemental Table 1). For intracellular staining, cells were first fixed in Fixation and Permeabilization Solution (BD Biosciences, catalog 554722) according to the manufacturer’s instructions. Cells were then stained with anti–TGF-β1–PE Ab, anti–γ-H2AX-APC Ab, or primary rabbit anti–mouse p16 Ab (Invitrogen, Thermo Fisher Scientific, catalog PA1-46220), followed by goat anti–rabbit-APC secondary Ab (Supplemental Table 1). For SPiDER–β-gal staining to identify SCs, live cells were stained with a cellular senescence detection kit (Dojindo, catalog SG04) following the manufacturers’ instructions. For the apoptosis assay, live cells were stained with anti–annexin V–FITC Ab and PI following the manufacturer’s instructions (BD Biosciences, catalog 556547). Cells were heated at 60°C as a positive control. Cells were subjected to flow cytometric analysis using a BD FACSCanto II Cytometer. Results were analyzed by Flowjo 7 data analysis software. For SC enrichment, callus cells were stained with rabbit anti–mouse p16 Ab (1:100, Invitrogen, Thermo Fisher Scientific, catalog PA1-46220), followed by goat anti–rabbit-PE and anti-PE Ab–conjugated microbeads (Miltenyi Biotec). p16^+^ SCs were magnetically enriched as previously described (72).

### shRNA knockdown.

The shRNA lentiviral particles targeting *Tgfb1* (sc-37192-V) and a scrambled sequence (sc-108080) were purchased from Santa Cruz Biotechnology. Infectious units of virus (10^5^ units) in DMEM with 2 mM HEPES, pH 7.3, were used for infection of the callus pieces that were harvested from young or aged mice on 10 dpf, following the manufacturer’s instructions. Fresh medium was changed, and callus pieces were cultured for another 2 days to generate CM. The efficiency of the lentivirus-mediated shRNA knockdown of *Tgfb1* was confirmed by qPCR.

### Quantitative real-time RT-PCR.

Fracture callus tissues were homogenized under liquid nitrogen. Total RNA was extracted using TRIzol reagent (Invitrogen, Thermo Fisher Scientific). cDNAs were synthesized using an iSCRIPT cDNA Synthesis Kit. Quantitative RT-PCR amplification was performed in an iCycler Real-Time PCR machine using iQ SYBR Green Supermix (all from Bio-Rad). β-Actin was amplified on the same plates and used to normalize the data. Each sample was prepared in triplicate, and each experiment was repeated at least 3 times. The relative abundance of each gene was calculated by subtracting the Ct value of each sample for an individual gene from the corresponding Ct value of β-actin (ΔCt). ΔΔCt was obtained by subtracting the ΔCt from the reference point. These values were then raised to the power 2 (2ΔΔCt) to yield the fold expression value relative to the reference point. Data are presented as the mean ± SD of the triplicates or of 4 wells of cell cultures. The primer sequences and qPCR conditions used are provided in Supplemental Table 2.

### Western blot analysis.

Fracture calluses were homogenized under liquid nitrogen. Proteins were extracted with RIPA lysis buffer. Proteins were quantitated using a kit from Bio-Rad and loaded onto 10% SDS-PAGE gels and blotted with anti–TGF-β1 Ab (1:100, Santa Cruz Biotechnology, catalog sc-130348), as described previously (72). Bands were visualized using ECL chemiluminescence (Bio-Rad, catalog 1705061).

### ELISA.

The levels of active TGF-β1 were measured using a mouse TGF-β1 DuoSet ELISA Kit (R&D Systems, catalog DY1679-05) following the manufacturer’s instructions. Absorbance in ELISA plates was read using a microplate reader set at 450 nm, with the wavelength correction set at 540 nm.

### Statistics.

All results are given as the mean ± SD. Statistical analysis was performed using GraphPad Prism 5 (GraphPad Software). The choice of statistical test was based on the numbers of groups and variables. Comparisons between 2 groups were analyzed using an unpaired, 2-tailed Student’s *t* test. One-way ANOVA with Tukey’s post hoc multiple comparisons was used for comparisons among 3 or more groups. Two-way ANOVA with Tukey’s post hoc multiple comparisons was used for analyses involving 2 variables. *P* values of less than 0.05 were considered statistically significant. The sample size for in vivo experiments was determined on the basis of our previous published data using 18-month-old aged mice with tibial fractures from which callus samples were harvested 2 weeks after drug treatments (35). The sample size (*n =* >3) was calculated according to the expected means of woven bone area 2.2 ± 0.4 mm^2^ in vehicle-treated mice, 3.5 ± 0.27 mm^2^ in drug 1–treated mice, and 4.0 ± 0.55 mm^2^ in drug 2–treated mice, with an α level of 0.05 and a power of 80%, using 1-way ANOVA with Dunnett’s test.

### Study approval.

All animal procedures were conducted in accordance with guidelines approved by the University of Rochester Committee for Animal Resources (protocol no. 2001-121R).

## Author contributions

JL, JZ, XL, BFB, HZ, and LX designed the experiments. JL, JZ, XL, and HZ conducted experiments and acquired data. JL, JZ, HZ, and LX wrote the original draft of the manuscript. All authors reviewed and edited the manuscript. The order of the co–first authors, JL and JZ, was determined by the number of experiments they performed.

## Supplementary Material

Supplemental data

## Version 1 04/15/2022

Electronic publication
