# Supplementary material for: Age-associated callus senescent cells produce TGF-β1 that inhibits fracture healing in aged mice
Source: J Clin Invest. 2022 Apr 15;132(8):e148073. doi: 10.1172/JCI148073 (PMC9012290; doi:10.1172/JCI148073)
Supplement: Supplemental data [file jci-132-148073-s110.pdf]

Supplemental Figures (10)
Supplemental Tables (2)

Supplemental Figure 1

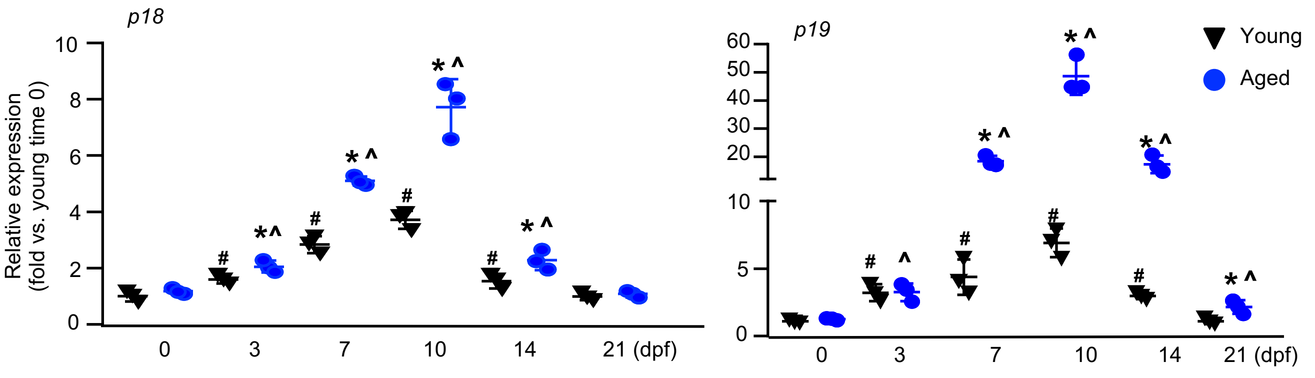

Supplemental Figure 1. Increased senescent cells in fracture callus of aged mice. Young and aged mice received tibial fracture surgery and were sacrificed on 3-, 7-, 10-, 14- and 21-dpf. (A) Expression levels of *p18* and *p19* mRNA in callus tissue examined by qPCR at the indicated time-points. n=3 mice/time-point. Relative expression is fold-change vs. young samples collected at time 0 as 1. Two-way ANOVA followed by Tukey post-hoc test. *p<0.05 aged vs. young; #p<0.05 young vs. young-time 0 (non-fractured bone); ^p<0.05 aged vs. aged-time 0 (non-fractured bone).

Supplemental Figure 2

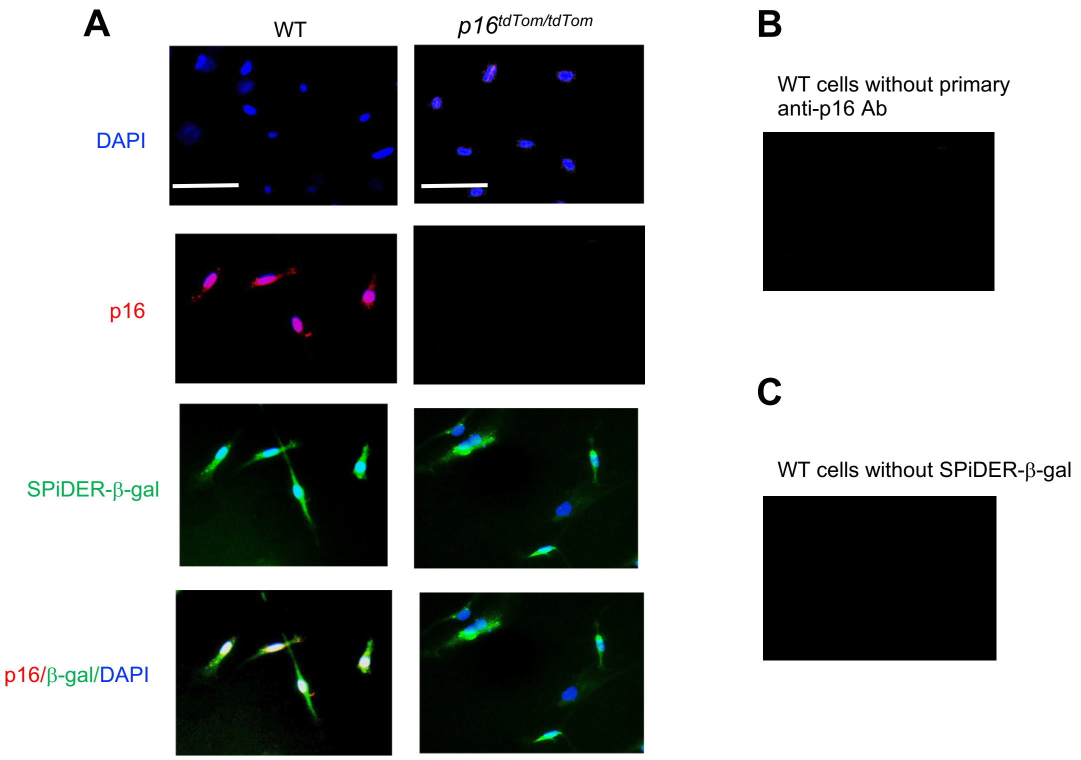

Supplemental Figure 2. To demonstrate the specificity of anti-p16 Ab. 3-m-old *p16*$^{dtTom/dtTom}$ (p16-deficient) and WT control mice at 10 dpf were used to generate CaMPCs. Cells were cultured on cover slices and treated H_2O_2 for 4 d to induce cellular senescence**. (**A) WT and *p16*$^{dtTom/dtTom}$ cells stained with a rabbit anti-mouse P16 Ab, followed by a goat anti-rabbit Alexa Fluor 568 secondary Ab, and for Spider-β-gal staining with a senescence β-gal staining kit. (B) WT cells stained with a goat anti-rabbit Alexa Fluor 568 secondary Ab (without anti-p16 Ab). (C) WT cells stained with staining solution without addition of SPiDER-β-gal reagent. Scale bar=50 mm. n=4 wells. Repeated once.

Supplemental Figure 3

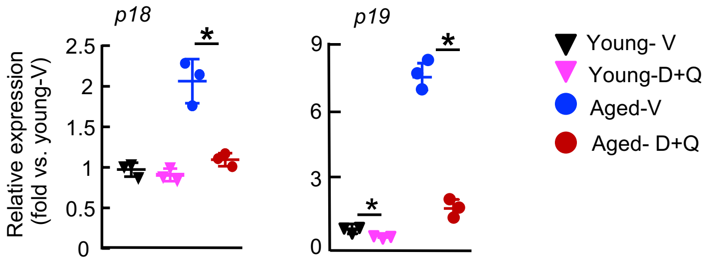

Supplemental Figure 3. Senolytic drugs decrease the expression of senescence genes in callus tissues from aged mice. Young and aged mice received tibial fracture surgery. Mice were given 5 mg/kg Dasatinib (D) + 50 mg/kg Quercetin (Q) or vehicle by gavage on 1-, 3-, 5-, and 7-dpf, and sacrificed at 10 dpf. Expression levels of *p18* and *p19* mRNA in callus tissues examined by qPCR. n=3 mice. Relative expression is fold-change vs. vehicle-treated young mice as 1. Two-way ANOVA followed by Tukey post-hoc test. *$p<0.05$ vehicle vs. D+Q treatment.

Supplemental Figure 4

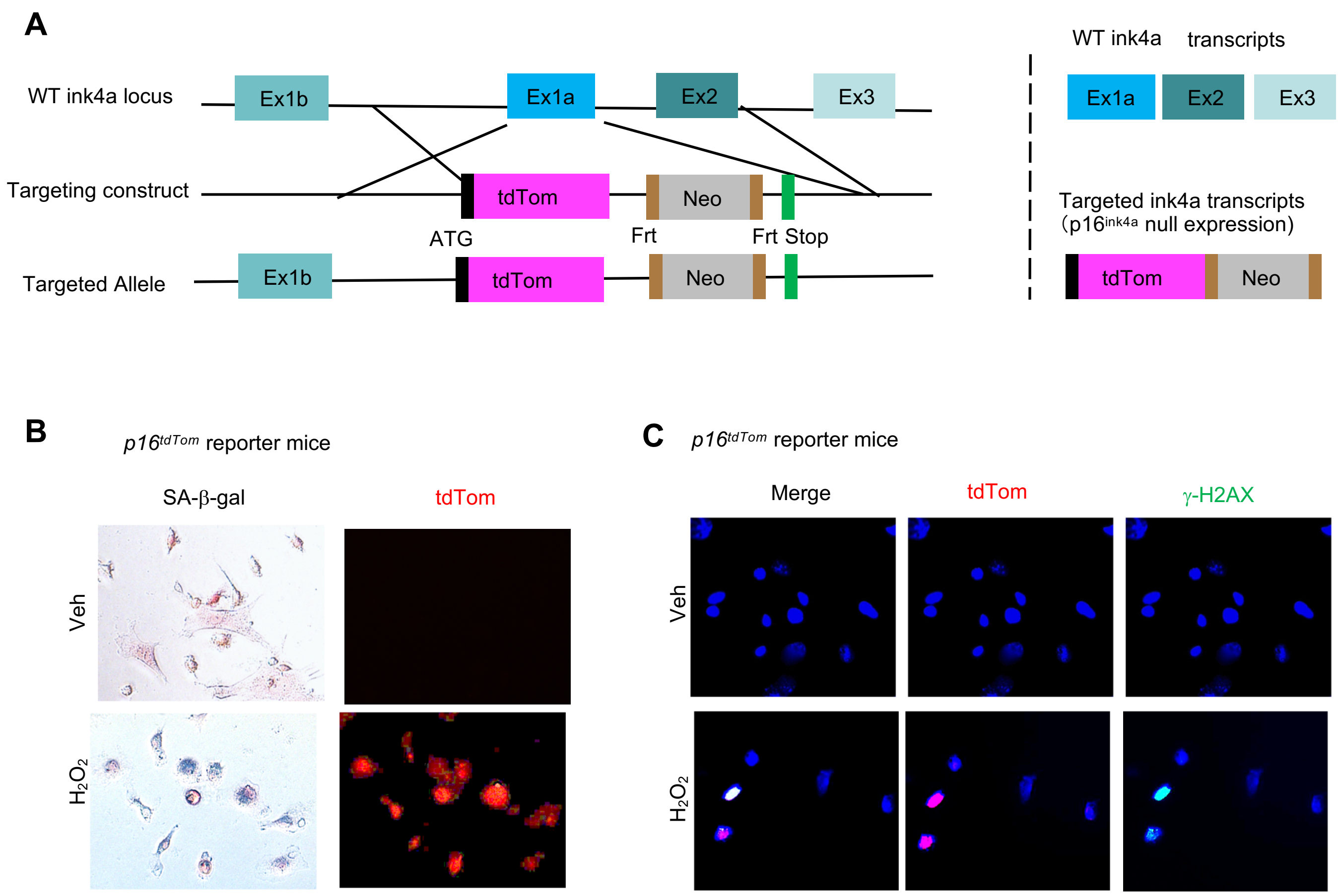

Supplemental Figure 4. Characterization of callus senescent cells using *p16^{tdTom}* reporter mice. Callus from 3-m-old *p16^{tdTom}* reporter mice 10 dpf were used to generate CaMPCs. (A) Schematic generation of *p16^{tdTom}* mice. Mice carrying one targeting allele used as *p16^{tdTom}* reporter mice. (B-C) 2nd passage of CaMPCs treated with vehicle (H_2O) or H_2O_2 for 4 d to induce cellular senescence. (B) SCs positive for tdTomato and SA-β-gal staining. (C) SCs positive for tdTomato and γ-H2AX staining. n=4 wells. Repeated once.

Supplemental Figure 5

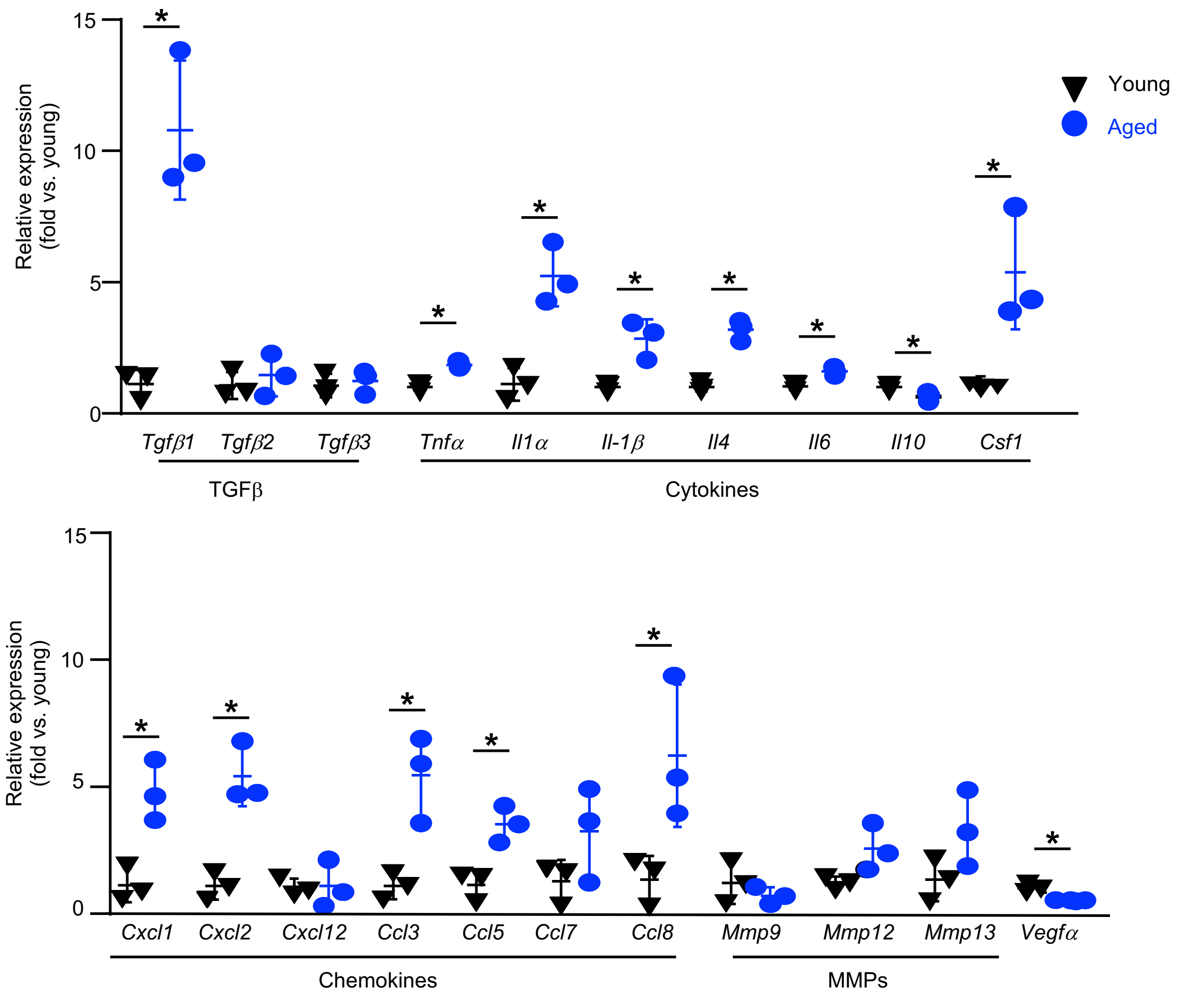

Supplemental Figure 5. Senescent cells in aged callus express high levels of TGFβ1. Young and aged mice were sacrificed at 10 dpf. Expression of SASP factors in callus tissues examined by qPCR. n=3 mice. Relative mRNA expression is the fold-change in aged samples vs. young samples as 1. Unpaired 2-tailed t-test, #$p<0.05$.

Supplemental Figure 6

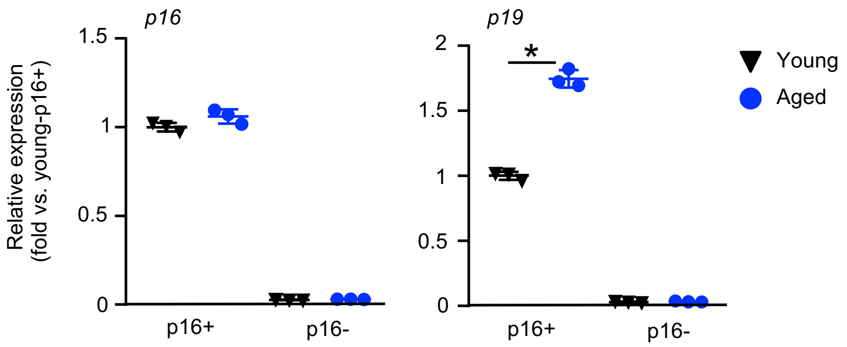

Supplementary Figure 6. Isolation of p16 cells from primary callus of mice with bone fracture. Young and aged mice received tibial fracture surgery and were sacrificed at 10 dpf. Callus tissues were cut into pieces and digested with Accumax cell detachment solution to generate cell suspensions. p16+ cells were isolated by indirect magnetic labeling. Callus cells were stained with a rabbit-anti mouse p16 Ab, followed by a PE conjugated anti-rabbit Ab. The cells were then incubated with anti-PE microbeads and passed through a MACS column to separate p16+ and p16- cells. Expression levels of the senescence genes, p16 and p19, measured by qPCR in p16+ and p16- cell populations. n=3 mice. Relative expression is the fold-change vs. p16+ cells isolated from young mice as 1. Unpaired 2-tailed t-tests. *$p<0.05$.

Supplemental Figure 7

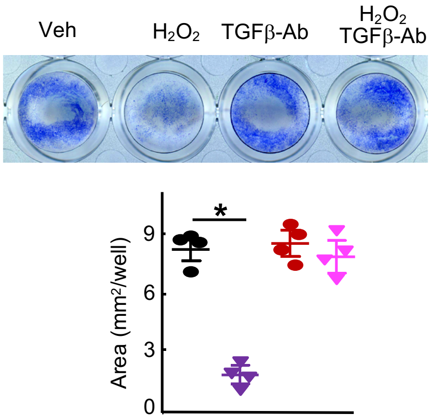

Supplemental Figure 7. TGFβ neutralizing Ab blocks the inhibitory effects of H_20_2–induced cell senescence on CaMPCs. Callus pieces were treated with H_2O_2 for 8 hr to induce cellular senescence and CM was collected. CaMPCs were treated with 30% CMs ± the TGFβ neutralizing Ab, 1D11, (1 μg/ml) or isotype IgG for 2 d. Cell growth assessed by methylene blue staining and positively-stained areas measured by Image J software. n=4 wells. Repeated once. One-way ANOVA followed by Tukey post-hoc test. *p<0.05 vehicle vs. other treatment.

Supplemental Figure 8

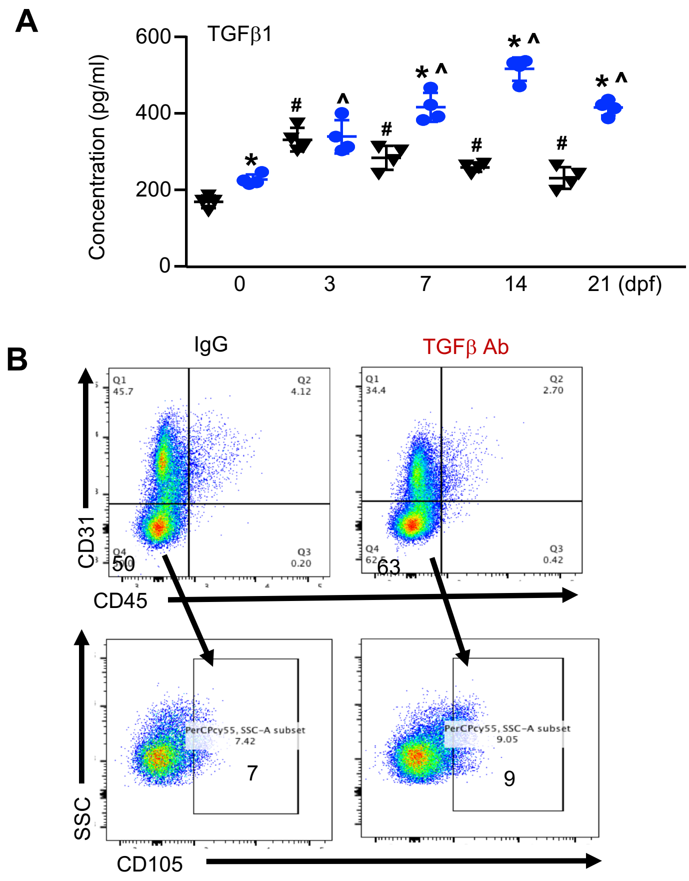

Supplemental Figure 8. TGFβ neutralization enhances callus volume in aged mice. (A) Young and aged mice received tibial fracture surgery. Concentration of active TGFβ1 protein in serum at indicated time-points measured by ELISA. n=4 mice. Two-way ANOVA followed by Tukey post-hoc test. *p<0.05 aged vs. young; #p<0.05 young vs. young-time 0 (non-fractured bone); ^p<0.05 aged vs. aged-time 0 (non-fractured bone). (B) Callus MPCs identified as CD45-/CD31-/CD105+ cells by flow cytometry.

Supplemental Figure 9

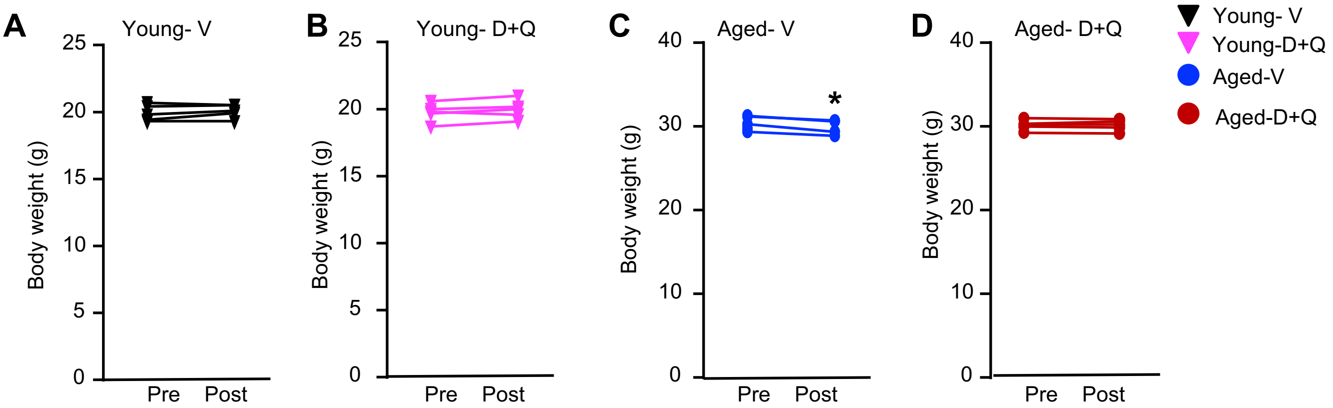

Supplemental Figure 9. Dasatinib plus quercetin treatment has no effect on body weight in aged mice. Young and aged female mice received tibial fracture surgery and treated with Dasatinib (D) plus Quercetin (Q), as indicated in Figure 8. Body weight was measured before and 14 dpf. (A-D) Body weight change pre- and post-treatment in young-V, Young- D+Q, Aged-V and Aged- D+Q group. Paired 2-tailed t-test. *p<0.05 vs. pre-treatment. n=4-5 mice.

Supplemental Figure 10

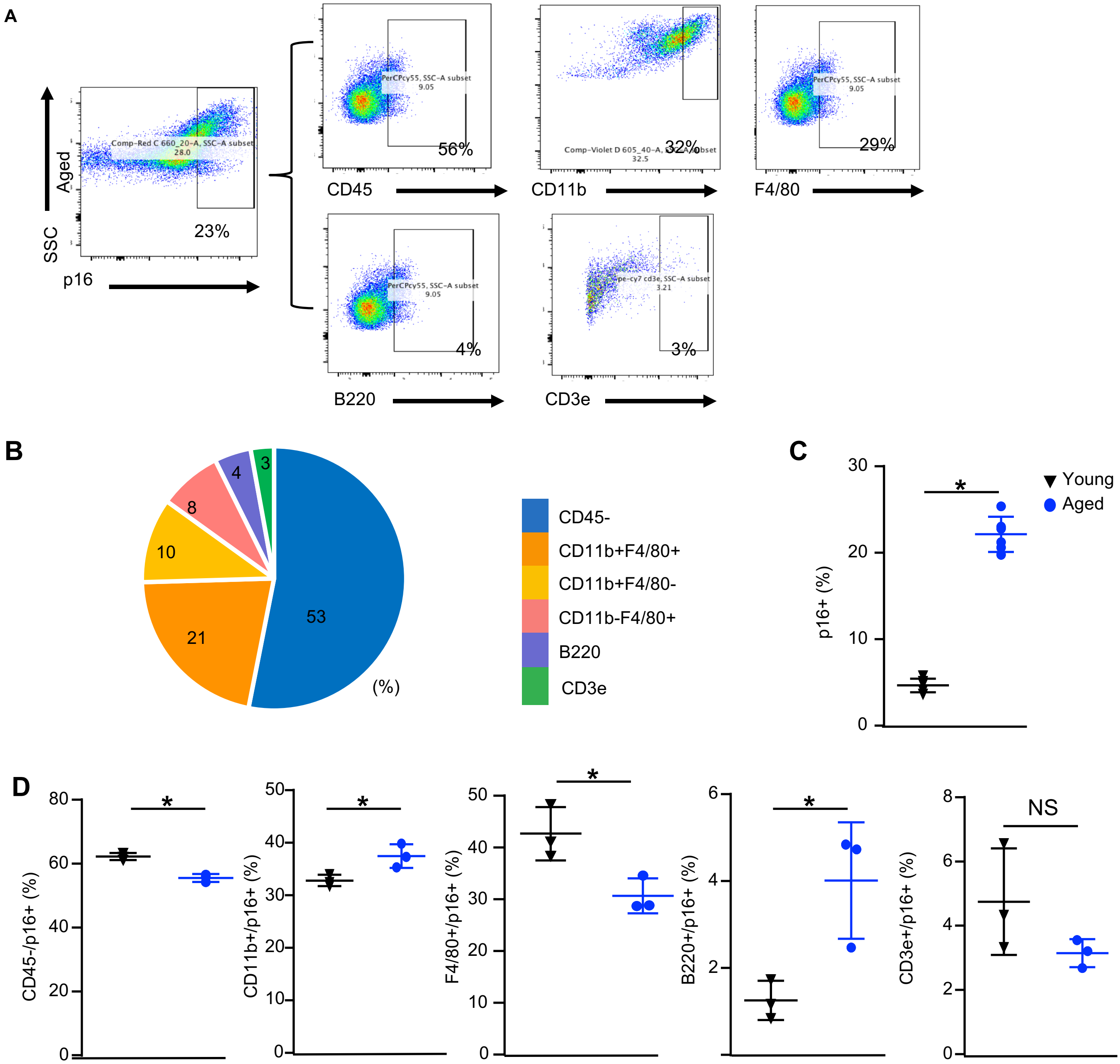

Supplemental data 10. Callus SCs consist of cells from both mesenchymal and myeloid lineages. Young and aged mice were sacrificed 10 dpf. Callus pieces were cultured for 2 d and subjected to flow cytometry. (A) p16+, CD45-, CD11b+, F4/80+, B220+ and CD3e+ cells from callus of aged mice identified by flow cytometry. (B) Relative percentage of CD45-, CD11b+F4/80+, CD11b+F4/80-, CD11b-F4/80+, B220+ and CD3e+ cells from callus of aged mice. (C) Percentage of p16+ cells. n=7 mice. (D) Percentage of CD45-, CD11b+, F4/80+, B220+ and CD3e+/p16+ cells from callus of aged mice versus young mice. n=3 mice. Statistical significance was calculated using a 2-tailed unpaired Student's t -test. "NS" means "not-significant". *p<0.05.

Supplemental table 1

Supplemental Table 1. Antibodies

Antibody	Clone	Species	Catalog #	eBiolegend	Dilution
CD44	IM7	mRt	17-0441	eBiolegend	1:100 (FACS)
CD105	MJ7/18	mRt	120415	eBiolegend	1:100 (FACS)
SCA1	D7	mRt	25-5981-82	eBiolegend	1:100 (FACS)
CD45	30-F11	mRt	25-0451-82	eBiolegend	1:100 (FACS)
CD45	30-F11	mRt	103108	eBiolegend	1:100 (FACS)
CD34	RAM34	mRt	11-0341-82	eBioscience	1:100 (FACS)
TER119	TER-119	mRt	12-5921-82	eBioscience	1:100 (FACS)
B220	RA3-6B2	mRt	103230	eBiolegend	1:100 (FACS)
CD31	MEC13.3	mRt	11-031-82	eBiolegend	1:100 (FACS)
CD31	MEC13.3	mRt	102515	eBiolegend	1:100 (FACS)
CD3e	145-2C11	mHm	100320	eBiolegend	1:100 (FACS)
F4/80	BM8	mRt	123128	eBiolegend	1:100 (FACS)
CD11b	M1/70	mRt	11-0112-41	eBiolegend	1:100 (FACS)
TGFβ1	TW4-9E7	mMs	562339	Invitrogen	1:100 (FACS)
p16	N/A	pRb	PA1-46220	Invitrogen	1:20 (IF); 1:50 (FACS)
gamma-H2AX	2F3	mMs	613415	eBiolegend	1:100 (FACS)
BrdU	Polyclonal	pRb	152095	Abcam	1:400 (IF)
F(ab) fragment anti-mouse IgG (H+L)	N/A	Goat	115-007-003	Jackson ImmunoResearch	1:400 (IF)
Gamma-H2AX Ab	JBW301	mMs	05-636-I	Sigma-Aldrich	1:250 (IF)
Ki67	N/A	pRb	ab15580	Abcam	1:400 (IF)
TGFβ1	3C11	mMs	sc-130348	Santa Cruz	1:100 (WB)
Actin	Polyclonal	pRb	A2066	Sigma-Aldrich	1:2000 (WB)

Supplemental table 2

Supplemental Table 2. Primers used in qPCR

Genes	Sequences of primers	GenBank accession #	Annealing Tm (℃)	Product (bp)
$p16^{Ink4a}$	F-5'-CGAACTCTTTCGGTCGTACCC-3'	NM_001040654.1	65	88
	R-5'-CGAATCTGCACCGTAGTTGAGC-3'			
$p19^{ARF}$	F-5'-GTTCTTGGTCACTGTGAGGATT-3'	NM_009877.2	63	194
	R-5'-CCATCATCATCATCACCTGGCCAG-3'			
beta-actin	F-5'-AGATGTGGATCAGCAAGCAG-3'	NM_007393.5	62	124
	R-5'-GCGCAAGTTAGGTTTTGTCA-3'			
Runx2	F-5'-CCTGAACTCTGCACCAAGTC-3'	NM_001145920.2	63	233
	R-5'-GAGGTGGCAGTGTCATCATC-3'			
Osx	F-5'-GTCAAGAGTCTTAGCCAAACTC-3'	XM_006520519.5	62	123
	R-5'-AAATGATGTGAGGCCAGATGG-3'			
Sox9	F-5'-CGGAACAGACTCACATCTCTCC-3'	NM_011448.4	63	162
	R-5'-GCTTGCACGTCGGTTTTG-3'			
Col2	F- 5’ GGGTCACAGAGGTTACCCAG-3’	NM_031163	60	85
	R- 5’ ACCAGGGGAACCACTCTCAC-3’			
Ppar-gamma	F-5'-GCCCTTTGGTGACTTTATGG-3'	XM_036165927.1	62	168
	R-5'-CAGCAGGTTGTCTTGGATGT-3'			
Cebpalpha	F-5'-CAAGAACAGCAACGAGTACCG-3'	NM_001287514.1	64	123
	R-5'-GTCACTGGTCAACTCCAGCAC-3'			
Tgf beta1	F-5'-GAGAAGAACTGCTGTGTGCG-3'	XM_036152883.1	61	133
	R-5'-GTGTCCAGGCTCCAAATATAG-3'			
Tgf beta2	F-5'-CAGGAGTGGCTTCACCACAAAG-3'	NM_009367.4	66	159
	R-5'-TGGCATATGTAGAGGTGCCATCA-3'			
Tgf beta3	F-5'-TCGACATGATCCAGGGACTG-3'	NM_009368.3	64	115
	R-5'-CCACTGAGGACACATTGAAACG-3'			
Tnfalpha	F-5'-AGTAGACAAGGTACAACCCATC-3'	NM_013693.3	61	181
	R-5'-CACACTCAGATCATCTTCTCAA-3'			
IL1alpha	F-5'-ATGATGGCTTATTACAGTGGCAA-3'	NM_000576.3	64	131
	R-5'-GTCGGAGATTCGTAGCTGGA-3'			
IL6	F-5'-AGTTGCCTTCTTGGGACTGA-3'	NM_001314054.1	60	158
	R-5'-TCCACGATTTCCCAGAGAAC-3'			
IL10	F-5'-CTATCCCTTGATGCCATTACCAG-3'	NM_008607.2	61	144
	R-5'-ATCCACATGGTTGGGAAGTTC-3'			
Csf1	F-5'-TGTCCTGTCACAAGCTCTGG-3'	XM_036162880.1	63	207
	R-5'-AGGGCATCAGAACAAGTTGG-3'			
Cxcl1	F-5'-GCTTGAAGGTGTTGCCCTCAG-3'	NM_008176.3	66	77
	R-5'-AAGCCTCGCGACCATTCTTG-3'			
Cxcl2	F-5'-GCGCTGTCAATGCCTGAAGA-3'	NM_009140.2	65	136
	R-5'-TTTGACCGCCCTTGAGAGTG-3'			
Cxcl12	F-5'-ATGAACGCCAAGGTCGTG-3'	NM_199168.4	62	220
	R-5'-CTTTAGCTTCGGGTCAATGC-3'			
Ccl3	F-5'-TGAAACCAGCAGCCTTTGCTC-3'	NM_011337.2	67	124
	R-5'-AGGCATTCAGTTCCAGGTCAGTG-3'			
Ccl5	F-5'-GACACCACACCCTGCTGCT-3'	NM_013653.3	65	62
	R-5'-TACTCCTTGATGTGGGCACG-3'			
Ccl7	F-5'-ACGCTTCTGTGCCTGCTGCTCATAG-3'	NM_013654.3	66	563
	R-5'-GTAAAAATGGGGAAAGGGGGAGAAT-3'			
Ccl8	F-5'-AGTGCTTCTTTGCCTGCTGCTCATAG-3'	NM_021443.3	70	388
	R-5'-ATGAGAAAACACGCAGCCCAGGCACC-3'			
Mmp9	F-5'-CCATGCACTGGGCTTAGATCA-3'	NM_013599.4	61	146
	R-5'-GGCCTTGGGTCAGGCTTAGA-3'			
Mmp12	F-5'-TTTCTTCCATATGGCCAAGC-3'	NM_001320077.1	62	197
	R-5'-GGTCAAAGACAGCTGCATCA-3'			
Mmp13	F-5'-CGGGAC61ATCCTGAAGAAGTCTACA	NM_008607.2	62	74
	R-5'-CTAAGCCAAAGAAAGATTGCATTTC-3'			
Vegf alpha	F-5'-GCACATAGAGAGAATGAGCTTCC-3'	NM_001110267.1	64	104
	R-5'-CTCCGCTCTGAACAAGGCT-3'			
Alp	F- 5’ CGGGACTGGTACTCGGATAA-3’	XM_006538498.4	56	157
	R- 5’ ATTCCACGTCGGTTCTGTTC-3’			
Trap	F-5’-TCCTGGCTCAAAAAGCAGTT-3’	XM_006509946.3	63	212
	R-5’-ACATAGCCCACACCGTTCTC-3’			
Ctsk	F-5’-CAGCTTCCCCAAGATGTGAT-3’	NM_007802.4	63	167
	R-5’-GAAGCACCAACGAGAGGAGA-3’			
